# Development of a 3D-Printed Two-Stage Virtual Impactor for Radioactive Aerosol Size Classification and Direct Analysis

**DOI:** 10.1021/acsomega.6c04521

**Published:** 2026-07-19

**Authors:** Hugo Laffolley, Youichi Tsubota, Ayame Kuroe, Tomoaki Kato

**Affiliations:** † Collaborative Laboratories for Advanced Decommissioning Science, 26300 Japan Atomic Energy Agency, 2-4 Shirakata, Tokai-Mura, Naka-Gun, Ibaraki 319-1195, Japan; ‡ Radiation Protection Department, Nuclear Cycle Engineering Laboratories, 26300 Japan Atomic Energy Agency, 4-33 Muramatsu, Tokai-Mura, Naka-Gun, Ibaraki 319-1194, Japan

## Abstract

Radioactive aerosol size classification is important for exposure assessment and decommissioning operations, particularly at the Fukushima Daiichi Nuclear Power Plant decommissioning site, where airborne particles may be generated or resuspended during remote inspection and debris retrieval. This study presents the development of a two-stage 3D-printed virtual impactor, called μSPLIT, designed to separate aerosols into three aerodynamic diameter classes (>10 μm, 1–10 μm, and <1 μm) while enabling direct postcollection analysis on integrated filters. The objective is to develop a virtual impactor that can be fabricated at a reduced cost and be disposed of easily after usage by incineration, without generating any hazardous human exposure during cleaning and decontamination activities, and free from metallic wastes that are not easily disposed of. The flow path was designed and optimized by computational fluid dynamics and Lagrangian particle tracking, and prototypes were fabricated by stereolithography. Numerical simulations predicted cutoff diameters of 9.0 and 1.3 μm for the first and second stages, respectively, with relatively sharp separation in both cases. The simulations also identified limitations of the current geometry, including small-particle contamination in the minor flow and large-particle contamination in the major flow of the second stage. Dimensional measurements of printed prototypes revealed contraction and corner rounding in the internal channels, confirming the need for fabrication compensation and quality control. Experimental tests with incense smoke provided a preliminary functional check, showing that submicrometric particles were mostly collected in the <1 μm particle class. Additional tests with Rn-progeny-bearing NaCl particles, characterized by a geometric mean aerodynamic diameter of 1.70 μm, produced the highest α activity on the middle filter (1–10 μm particle class), consistent with the expected classification. These results demonstrate the proof of concept of a lightweight and low-cost impactor for simultaneous aerosol size classification and direct radioactive particle analysis, while also identifying key areas requiring further optimization, particularly the second-stage geometry, quantitative wall loss assessment, and validation with standardized aerosols.

## Graphical abstract

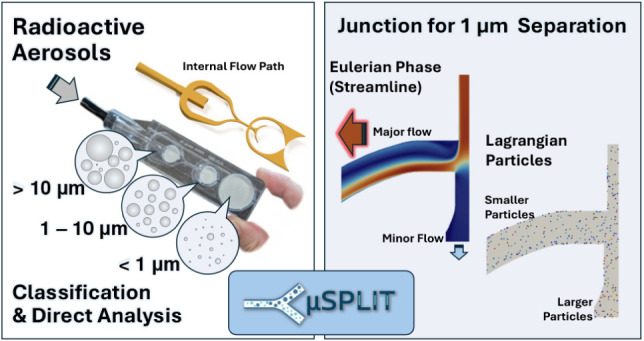

## Introduction

The assessment of radioactive aerosol size distribution is crucial to radiation protection and environmental monitoring in nuclear facilities. Following a large-scale survey conducted on the activity median aerodynamic diameter (AMAD) of aerosols in workplaces, a value of 5 μm was determined as the most realistic default value for occupational exposure estimation and later often used as a reference value for exposure assessment.^1^ However, the penetration depth of an aerosol particle in the respiratory system is a direct function of its aerodynamic diameter, with micrometric particles more likely to deposit in the extrathoracic region and submicrometric particles more likely to deposit in the bronchiolar and alveolar regions.^2^ Consequently, the effective dose delivered by 100 nm particles relative to that of the default 5 μm particles is estimated to be approximately 3.5 times higher for ^234^U and ^239^Pu, respectively.^1,3^ Furthermore, size distribution greatly influences dispersion and deposition patterns as well as resuspension of aerosol particles.^4,5^ In nuclear decommissioning contexts, this information becomes even more crucial as it directly influences decision-making regarding worker safety protocols and containment strategies.

Following the 2011 accident at the Fukushima Daiichi Nuclear Power Plant (1F), which caused core melting in three reactors,^6^ the decommissioning project presents a challenging environment for radioactive aerosol hazard management. The high ambient dose rate near the containment vessels of the damaged reactors has led to limiting worker exposure through the use of remotely controlled means for situation evaluation, activity monitoring, and conducting preliminary decommissioning operations such as core fragment retrieval.^7^ Among other methods, flying drones are deployed inside the reactor buildings by the operator TEPCO (Tokyo Electric Power Company) to investigate, observe, and collect data.^8^ During the accident progression, the reactor pressure vessels of the damaged reactors failed, and leaks from the containment vessels occurred, leading, for example, to hydrogen explosions.^9^ Consequently, a certain amount of radioactive aerosol is estimated to be deposited on exposed surfaces throughout the reactor buildings and could be resuspended during drone operations. This could significantly worsen air quality inside the buildings and cause leaks to the environment due to a loss of building integrity. In addition, the massive amount of molten fuel and structural debris requires cutting operations for extraction. Currently, TEPCO is considering several mechanical and thermal cutting methods that will inevitably generate highly radioactive aerosols. Some studies have shown that the cutting method, atmospheric conditions, and material composition (intrinsically heterogeneous) greatly influence aerosol size distribution over a wide range of aerodynamic diameters.^10,11^

Several techniques have proven effective in classifying aerosol particles according to size, with inertial methods being most prevalent due to their operational simplicity relative to alternatives such as electrical mobility classifiers. Inertial approaches vary in design but often use particle drag characteristics, typically making them passive systems (with the exception of the pumps).^5^ These simplified devices, without any moving parts, show particular suitability for harsh environments such as 1F buildings. Among inertial separation methods, virtual impaction (VI) presents advantages over centrifugal separation because of its characteristically sharper particle size cutoff and generation of two discrete, still-airborne effluent streams downstream of separation.^12,13^

Traditional virtual impactors have typically been fabricated from metals such as stainless steel, aluminum, or brass to ensure dimensional precision, mechanical durability, and chemical resistance.^14^ This provides excellent performance characteristics including smooth internal surfaces that minimize particle losses, thermal stability for high-temperature applications, and resistance to corrosive species. However, these advantages come with some practical limitations. Metal virtual impactors require thorough cleaning after each use to prevent cross-contamination between samples and optimal performance. This cleaning procedure would be a labor-intensive process leading to an extensive external radiation exposure to the workers from the deposited particles and during the recovery process, and a high risk of internal contamination during the disassembly and cleaning. In the context of 1F decommissioning, such tasks are avoided and cannot become a routine activity. Weight is also a consideration, since monitoring at specific locations may be performed by a quadruped robot. Furthermore, the manufacturing process for metal impactors involves machining operations that limit design flexibility and make rapid prototyping or customization prohibitively expensive.

In response to these limitations, this study proposes a novel 3D-printed virtual impactor designed specifically for radioactive aerosol characterization in challenging environments like the 1F decommissioning site. Such development has already been achieved for other purposes and under different operating conditions.^15^ The aim is to develop a two-stage virtual impactor to split particle-laden airflow into three particle size classes in terms of aerodynamic diameter: larger than 10 μm (class A), between 1 and 10 μm (class B), and smaller than 1 μm (class C). The additive manufacturing approach offers numerous advantages over traditional metal fabrication methods. First, 3D-printed virtual impactors can be produced at a fraction of the cost of metal equivalents, making them economically viable as single-use disposable devices that eliminate cross-contamination concerns and cleaning requirements. Second, the polymer materials used in 3D printing result in significantly lighter weight devices that are easier to handle and deploy. Third, the design flexibility afforded by additive manufacturing allows for rapid prototyping and optimization of impactor geometry to achieve specific classification characteristics, as well as the integration of additional features that enhance functionality. In addition, our 3D printed virtual impactor has been designed to enable direct analysis of collected particles without transfer steps. The structure incorporates features that allow for in situ radioactivity measurement using radiation detectors, especially α-containing particles, and potentially direct chemical analysis through techniques such as X-ray fluorescence or laser-induced breakdown spectroscopy. This integrated approach minimizes sample handling, reduces potential exposure to personnel, and provides more accurate characterization of radioactive aerosols by avoiding losses during transfer between instruments. The development of this 3D printed virtual impactor also aims to provide a cost-effective solution for the aerosol science community.

## Materials and Methods

### Virtual Impactors

#### Description

Traditional inertial impactors achieve particle size separation by accelerating aerosol particles through a converging nozzle toward a solid collection plate. Larger particles have sufficient inertia, overcome aerodynamic drag and impact upon the plate. Smaller particles follow the gas streamlines around the impaction point and are carried away in the exhaust flow. The virtual impactor is a modification of this prior design.^14^ It eliminates the solid impaction surface, substituting it with a controlled flow split at the nozzle exit plane. A small suction stream (minor flow) is set to align with the trajectory of large particles exiting the nozzle. Due to their momentum, these large particles cross streamlines and are captured in the minor flow. Smaller particles, lacking sufficient inertia to deviate significantly, remain within the major exhaust flow stream (Figure 1).

**Figure 1:**
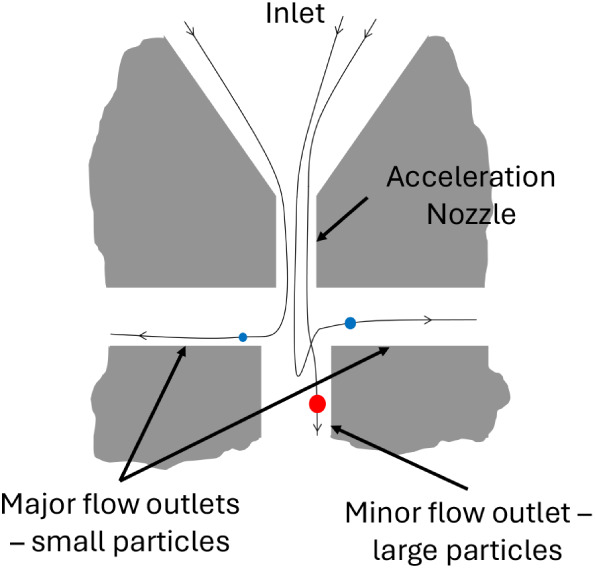
Schematic cross-section of a simple virtual impactor. The particle-laden airflow enters through the top inlet and is directed through the acceleration nozzle to establish the required flow velocity. A large fraction of the inlet flow is directed to the major flow outlets (e.g., 90%). Small particles (blue) follow the streamlines and are mostly directed toward the major outlets, while larger particles (red), due to their higher inertia, continue along the axial direction and enter the minor flow.

#### Design

For this study, the development has been based on the theoretical design of an optimized virtual impactor and results from previous development of a low flow rate 3D printed and flat virtual impactor.^15–18^ The following equation serves as a design reference:

1 $$d_{50}=\sqrt{\frac{18\mu w_{nozzle}Stk_{50}}{C\rho_{p}{\mathrm{u̅}}_{nozzle}}}$$

where *d*_50_ is the particle cutoff diameter, μ the air viscosity, *w*_*nozzle*_ the width of the acceleration nozzle, ρ_*p*_ the particle density, *u̅*_*nozzle*_ the average air velocity in the acceleration nozzle, *C* the Cunningham slip correction factor and *Stk*_50_ the Stokes number also referred as the particle inertia parameter for particles with a diameter equal to *d*_50_.

Empirical results suggest that a *Stk*_50_ greater than 1 improves separation efficiency for this kind of design and without any clean air addition.^17^

The Cunningham slip correction factor can be defined by^5^

2 $$C=1+\frac{\lambda }{d_{p}}\left[2.34+1.05\exp\left(-0.39\frac{d_{p}}{\lambda }\right)\right]$$

where *d*_*p*_ is the diameter of the particle and λ the air mean free path.

### Numerical Simulation

#### Approach

With respect to the parameters defined previously and the objective of creating a pseudo two-dimensional virtual impactor, a draft design of the flow path was developed. The design was then refined through iterative numerical simulations and design corrections. The numerical simulations were conducted in two steps: first, the steady-state turbulent Eulerian flow was solved; then, the transient simulations of the Lagrangian particle trajectories were computed on the previously calculated flow field. This means that only the Eulerian phase affects the Lagrangian phase. This is referred to as one-way coupling and is considered valid for a volume fraction of particle below 10^–6^.^19^ This work aims to design a virtual impactor that separates the particles into three aerodynamic size-related classes, meaning that this virtual impactor consists of two junctions, or separation stages. The numerical simulations of the two stages have been carried out separately to reduce the computational cost and optimize them on two-dimensional geometries. The work was conducted using OpenFOAM v2412.

#### Geometry and Domain

Figure 2 shows the two-dimensional geometry of the fluid subdomains used for each junction case at the beginning of the numerical simulations, prior to any design optimization. A two-dimensional subdomain consists of an inlet, a major outlet with a 90° deviation, a minor flow outlet facing the inlet, a symmetry boundary, and walls. The initial estimates for the widths of the acceleration nozzle, the major flow channel, and the minor flow channel were made in accordance with the recommendations developed earlier.

**Figure 2:**
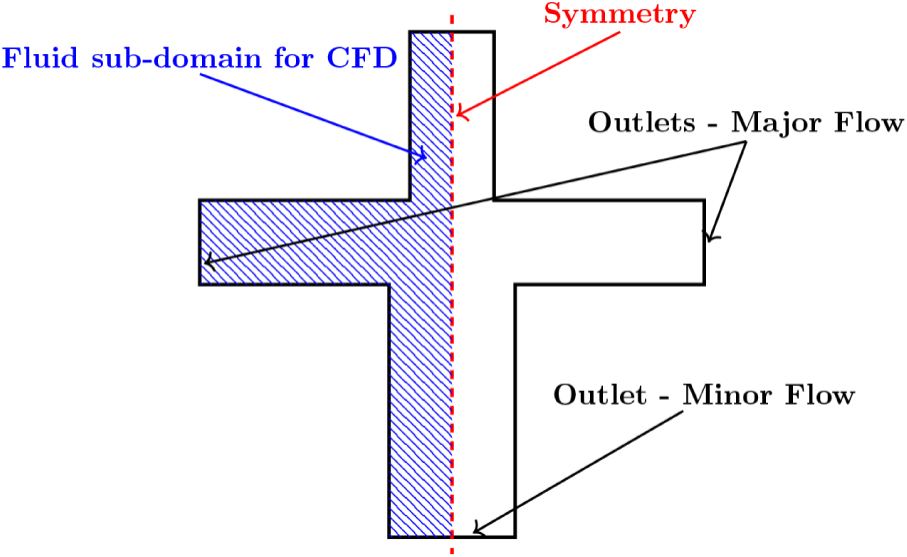
Geometry of the two-dimensional fluid subdomain (hashed blue) at the initial stage of numerical simulation, before any design optimization.

#### Operating Conditions

With regard to the predefined operating conditions, the volumetric flow rates are set to 10 L/min at the inlet, and to 1 L/min, 1 L/min, and 8 L/min at the class-A, class-B, and class-C outlets, respectively (at normal conditions of 273.15 K and 101,325 Pa). In the numerical simulations, the flow rates were specified as mass flow rates at the outlets, while the inlet flow rate was allowed to adjust freely and monitored to ensure mass conservation. The pressure was set at the inlet to 101,325 Pa and 95,000 Pa for the 10 and 1 μm junctions, respectively. The value of 95,000 Pa was estimated to account for upstream pressure losses. The working fluid was modeled as a compressible pure gas mixture representing air, initially at 300 K. A compressible gas model is required because the flow reaches *Ma* = 0.5 in the 1 μm separation stage subdomain. For consistency, the same models and solvers were used for both subdomains. The discrete phase was modeled as spherical particles with a density ρ_*p*_ = 1000 kg m^–3^, allowing a direct reading of the aerodynamic diameter, and injected at the inlets. The transient simulation lasted 20 ms, during which a total of 1 × 10^6^ particles were injected over the first 10 ms. The particle size distribution was uniform, ranging from 500 nm to 50 μm and from 100 nm to 10 μm for the 10 and 1 μm separation stages, respectively. Additional details on the fluid modeling properties and the injected particles size distribution are provided in the Supporting Information S1 and S2.

#### Governing Equations of the Eulerian Flow

The governing equations are the steady-state, compressible Reynolds-Averaged Navier–Stokes (RANS) equations for a gas mixture, closed with the *k*–ω shear stress transport (SST) turbulence model. A density-weighted (Favre) averaging procedure is employed for the velocity field to account for compressibility effects. The resulting equations solved by the rhoSimpleFoam solver are^20^

3 $$\frac{\partial }{\partial x_{j}}\left(\rho {ũ}_{j}\right)=0$$

4 $$\frac{\partial }{\partial x_{j}}\left(\rho {ũ}_{i}{ũ}_{j}\right)=-\frac{\partial \mathrm{p̃}}{\partial x_{i}}+\frac{\partial }{\partial x_{j}}\left[\left(\mu +\mu_{t}\right)\left(\frac{\partial {ũ}_{i}}{\partial x_{j}}+\frac{\partial {ũ}_{j}}{\partial x_{i}}-\frac{2}{3}\delta_{ij}\frac{\partial {ũ}_{k}}{\partial x_{k}}\right)\right]$$

5 $$\frac{\partial }{\partial x_{j}}\left(\rho {ũ}_{j}{\mathrm{h̃}}_{t}\right)=\frac{\partial }{\partial x_{j}}\left[\left(\lambda +\frac{c_{p}\mu_{t}}{Pr_{t}}\right)\frac{\partial \mathrm{T̃}}{\partial x_{j}}\right]$$

where ρ is the density, *ũ*_*i*_ the Favre-averaged velocity components, *p̃* the mean pressure, *h̃*_*t*_ the total enthalpy, μ the molecular viscosity, μ_*t*_ the eddy viscosity, λ the thermal conductivity, and *Pr*_*t*_ the turbulent Prandtl number. The thermodynamic closure is provided by the ideal-gas equation of state for the mixture,

6 p̃=ρRT

with *R* the mixture-specific gas constant.

The turbulence closure is obtained from the *k*–ω SST model of Menter, which solves transport equations for the turbulent kinetic energy *k* and the specific dissipation rate ω:

7 $$\frac{\partial }{\partial x_{j}}\left(\rho {ũ}_{j}k\right)=P_{k}-\beta^{*}\rho k\omega +\frac{\partial }{\partial x_{j}}\left[\left(\mu +\sigma_{k}\mu_{t}\right)\frac{\partial k}{\partial x_{j}}\right]$$

8 $$\frac{\partial }{\partial x_{j}}\left(\rho {ũ}_{j}\omega \right)=\alpha \frac{\omega }{k}P_{k}-\beta \rho \omega^{2}+\frac{\partial }{\partial x_{j}}\left[\left(\mu +\sigma_{\omega }\mu_{t}\right)\frac{\partial \omega }{\partial x_{j}}\right]+2\left(1-F_{1}\right)\rho \sigma_{\omega 2}\frac{1}{\omega }\frac{\partial k}{\partial x_{j}}\frac{\partial \omega }{\partial x_{j}}$$

where *P*_*k*_ is the production of turbulent kinetic energy, and α, β, β*, σ_*k*_, σ_ω_, and σ_ω2_ are model constants. The blending function *F*_1_ ensures a smooth transition between the *k*–ω formulation near walls and the *k*–*ε* formulation in the free stream. The eddy viscosity is computed as

9 $$\mu_{t}=\frac{\rho a_{1}k}{\max\left(a_{1}\omega ,SF_{2}\right)}$$

where *S* is the strain-rate magnitude, *F*_2_ a second blending function, and *a*_1_ a model constant.

#### Lagrangian Particle Model

The dispersed phase was described with a Lagrangian parcel approach using a slightly modified version of the uncoupledKinematicParcelFoam solver.^21^ Initially, this solver considers a constant fluid density, but the source code has been modified to employ the imported density field calculated at the first step by the steady-state compressible solver rhoSimpleFoam. Parcels represent groups of identical spherical particles of diameter *d*_*p*_ and material density ρ_*p*_. One-way coupling was assumed, i.e., particle motion was influenced by the carrier phase but without feedback to the gas. Gravitational effects were omitted. Particle trajectories are governed by

10 $$\frac{dx_{p}}{dt}=v_{p}$$

11 $$\frac{dv_{p}}{dt}=F_{D}$$

where **x**_*p*_ and **v**_*p*_ are the parcel position and velocity vectors, respectively. Only drag forces were considered. The drag acceleration applied to the particle is

12 FD=34μcCDRepρpdp2urel

with μ_*c*_ the carrier-phase viscosity, ρ_*p*_ the particle material density, *d*_*p*_ the particle diameter, and **u**_rel_ = **u**-**v**_*p*_ the relative velocity between fluid and particle. The particle Reynolds number is defined as

13 Rep=ρc|urel|dpμc

where ρ_*c*_ is the local carrier-phase density. The drag coefficient is evaluated according to the correlation implemented in OpenFOAM:^22^

14 CD={24Rep(1+16Rep2/3),Rep≤10000.424,Rep>1000

Brownian motion was neglected in the particle trajectory model. An order-of-magnitude estimate using Einstein’s relation $\left(x_{\mathrm{rms}}=\sqrt{2Dt}\right)$ yields an RMS displacement of approximately 0.2 μm for 1 μm particles at the relevant flow time scale (∼0.001 s), which is an order of magnitude smaller than the cutoff diameter of the second stage (1.3 μm). Therefore, the contribution of Brownian diffusion to particle trajectory deviation was considered negligible.

### Prototyping

The prototypes were designed using Autodesk Fusion software, considering the specifications imposed by the numerical simulations, size constraints, and the requirement for real-time activity measurement. They were fabricated by stereolithography (Formlabs Form 4) using a transparent resin (Formlabs Clear V5) to enhance UV curing and enable internal visualization. After printing, postprocessing consisted of an agitated isopropanol bath (Formlabs Form Wash) for 10 min, followed by heated UV curing (Formlabs Form Cure) for 15 min at 60°C.

## Experimental Section

### Approach

Prior to further optimization or detailed experimental qualification of the μSPLIT, proof-of-concept experiments were conducted. The objective was to verify that the μSPLIT can effectively classify aerosols by size and that real-time activity measurement is feasible.

First, the μSPLIT was qualitatively evaluated for its ability to classify particles alone, by collecting soot particles generated from the combustion of incense sticks. A second protocol was then established to generate α-emitting particles from ^222^Rn progeny attached to NaCl particles, which were subsequently measured using a pixelated α-detector.

### Incense Smoke

An incense stick was ignited inside a 0.2 m^3^ volume, closed but not airtight, and the smoke was allowed to accumulate (Figure 3). After 5 min, the particle concentration was checked, and the initial particle size distribution was measured using a particle size spectrometer based on inertial impaction (Dekati ELPI+). Experiments were initiated once the particle number concentration within the combustion volume reached approximately 1 × 10^7^ cm^–3^.

**Figure 3:**
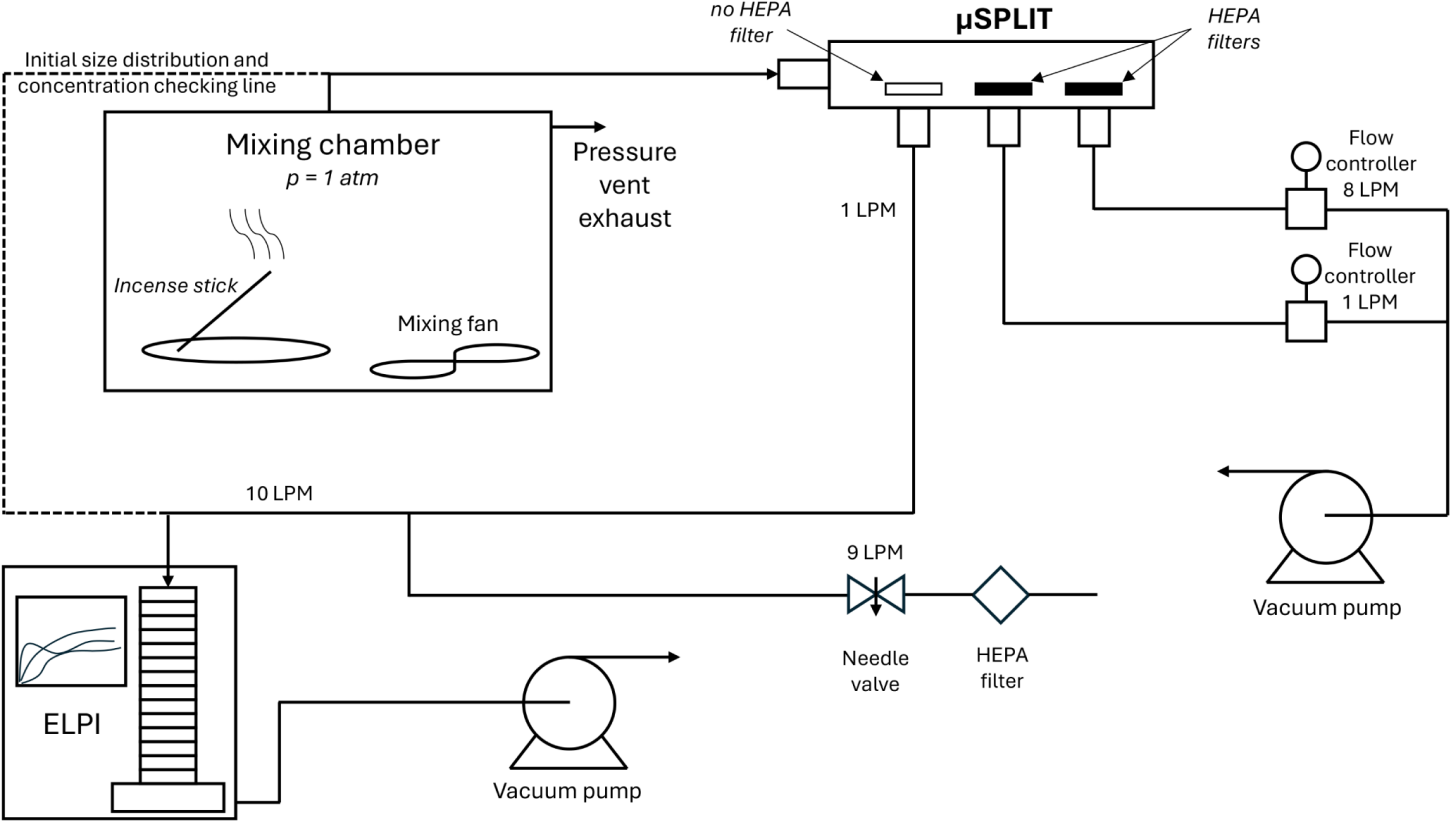
Diagram of the experimental setup for the generation, collection and measurement of the performance of the μSPLIT with incense smoke.

A μSPLIT was then set up, with its inlet connected to the combustion chamber. One outlet was connected to the particle spectrometer, while the two remaining outlets were respectively connected to a pump and fitted with a HEPA filter at the designated locations. Flow rates were controlled either by a mass flow controller or a control valve, following precalibration. The protocol was repeated to measure the size distribution at each outlet.

### ^222^Rn-NaCl Aerosol

Droplets were generated by a nebulizer from a 1% NaCl aqueous solution (Figure 4). The droplets were entrained into a 0.2 m^3^ airtight mixing volume by clean, dry air, causing the water to evaporate and form solid NaCl particles. In parallel, clean, dry air was passed through a water bubbler for rehumidification, then through a small box containing natural uranium-bearing rocks, before entering the mixing volume. This process caused the airborne progeny of ^222^Rn to attach to the NaCl particles, forming radioactive solid particles.

**Figure 4:**
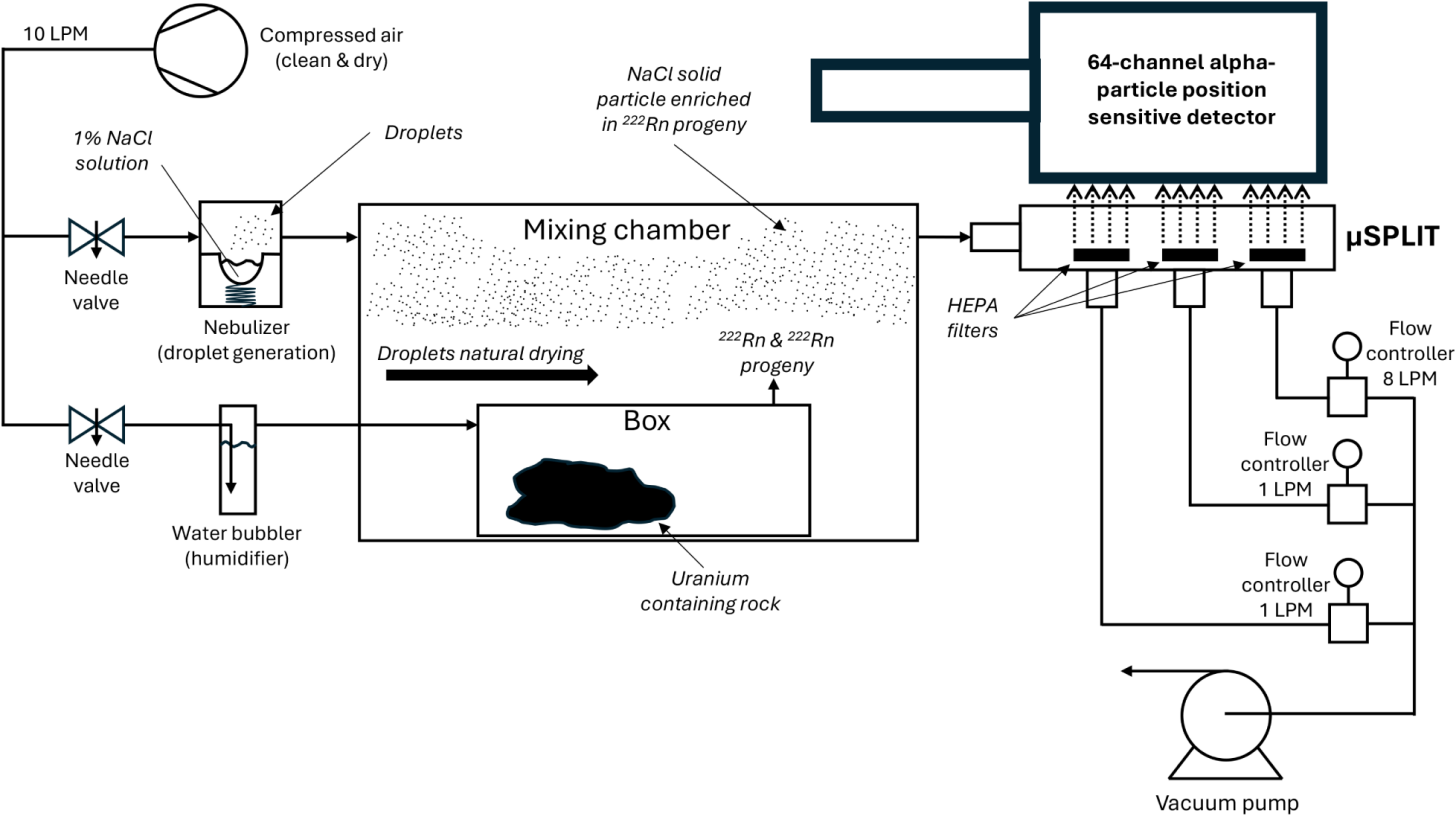
Diagram of the experimental setup for the generation, collection and measurement of the performance of the μSPLIT with Rn-progeny NaCl particles.

The total air flow rate was 10 L min^–1^. The particle-laden airflow was then directed to the μSPLIT, onto which HEPA filters were installed at the designated locations. Flow rates were set according to specifications using flow controllers and maintained by a vacuum pump. The amount of particles accumulated on the filters was monitored using a 64-channel α-particle position-sensitive detector, directly mounted on the μSPLIT in contact with the Mylar film.^23,24^

## Results and Discussions

### Design Optimization by Numerical Simulations

#### Geometry Specifications

Figure 5a shows the final design obtained after several CFD optimization steps. The internal design was selected to approach the desired cutoff diameters, and simple optimizations were implemented to improve sharpness and reduce the overall size. Figure 5b represents the internal hollow volume of the μSPLIT (i.e., the fluid domain), excluding the regions dedicated to supporting the HEPA filters. Figure 5c and 5d show the 2D subdomains optimized by CFD calculations for the two junction specifications.

**Figure 5:**
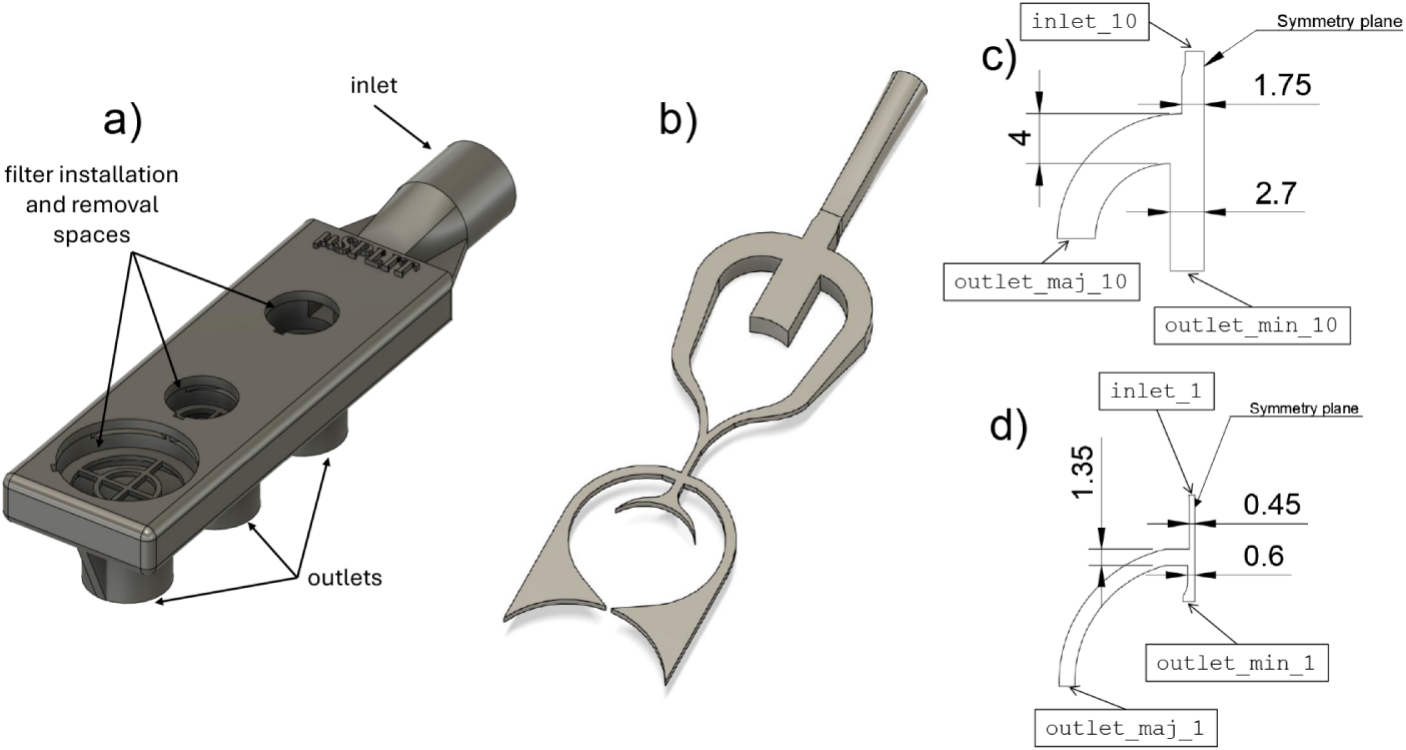
(a) 3D CAD model of the μSPLIT. (b) View of the fluid domain inside the μSPLIT. (c) and (d) 2D fluid subdomains of the 10 and 1 μm cutoff diameter junctions used for 2D numerical simulations.

The calculations indicate that, for these design choices, the acceleration nozzle of the 10 μm junction should have a cross-section of 12.25 mm^2^ (3.5 × 3.5 mm), while the 1 μm junction should have a cross-section of 0.9 mm^2^ (1.0 × 0.9 mm).

#### Eulerian Phase

Figure 6 shows the velocity field and streamlines of the Eulerian phase at the 10 μm cutoff diameter junction. The flow enters from the top inlet patch, passes through the acceleration nozzle (≈12 m/s), and bifurcates at the junction into the major flow, which is deflected by 90° into the curved channel, and the minor flow, which continues axially downward. The velocity peak observed at the inner corner of the junction is consistent with the centrifugal acceleration of the fluid as it follows the sharp bend, and defines the local aerodynamic conditions governing inertial particle classification.

**Figure 6:**
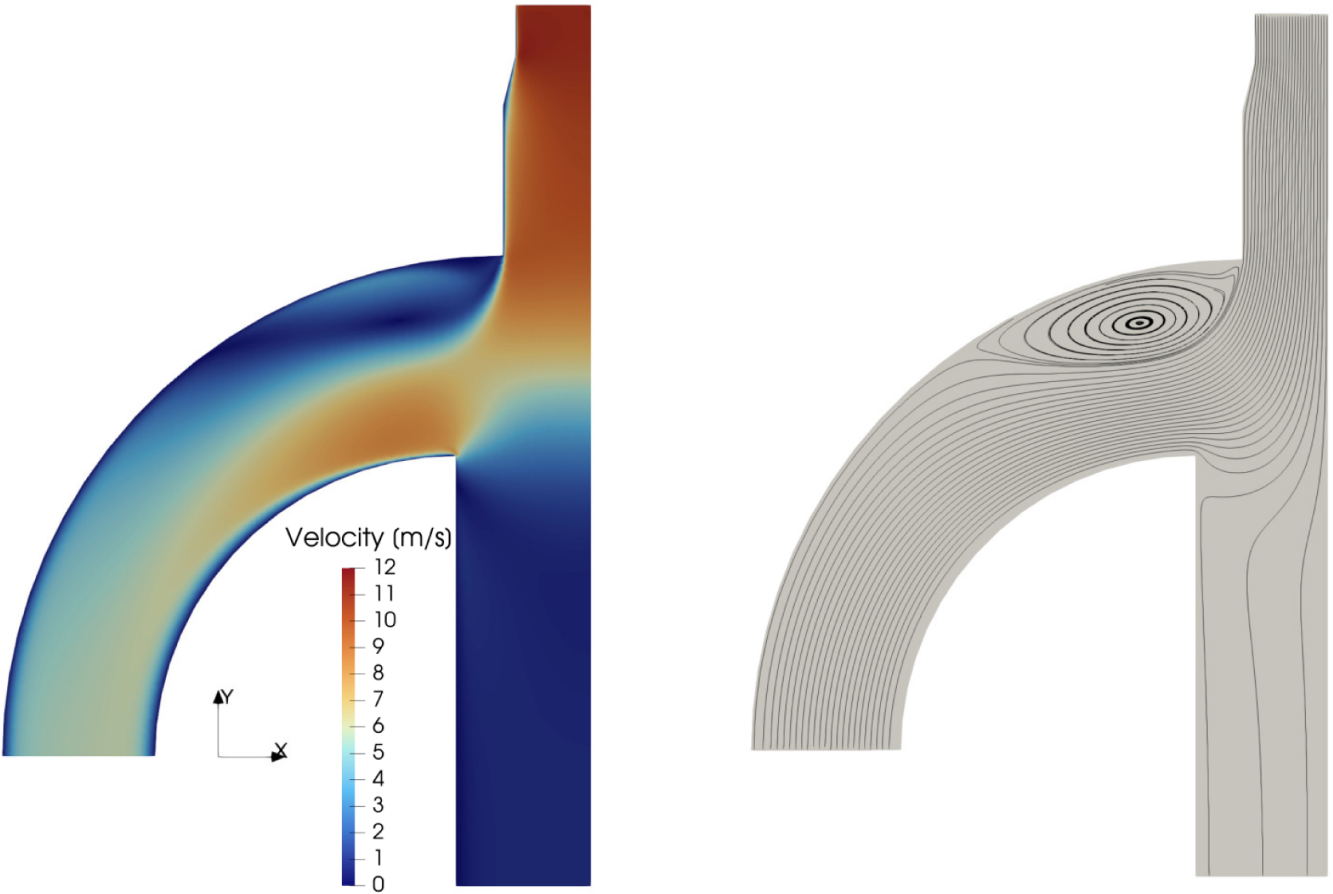
(left) Velocity field and (right) flow streamlines of the 10 μm cutoff diameter junction.

The minor flow channel shows near-stagnant conditions, confirming the intended low minor-to-major flow ratio and validating the virtual impactor operating regime. Streamline analysis reveals a stable recirculation eddy at the inner corner of the junction, resulting from flow separation at the abrupt geometric transition. This recirculation may locally perturb the pressure balance at the bifurcation, cause additional wall losses, and promote particle recirculation.

The observations made at the 1 μm junction are largely similar to those at the 10 μm junction (Figure 7). However, a significant difference is observed in the fluid velocity, which reaches approximately 150 m/s (*Ma* ≈ 0.5) in the acceleration nozzle and in the inner region of the major flow curved channel near the entrance. This confirms the need for a compressible approach in the numerical simulation to account for variations in fluid density.

**Figure 7:**
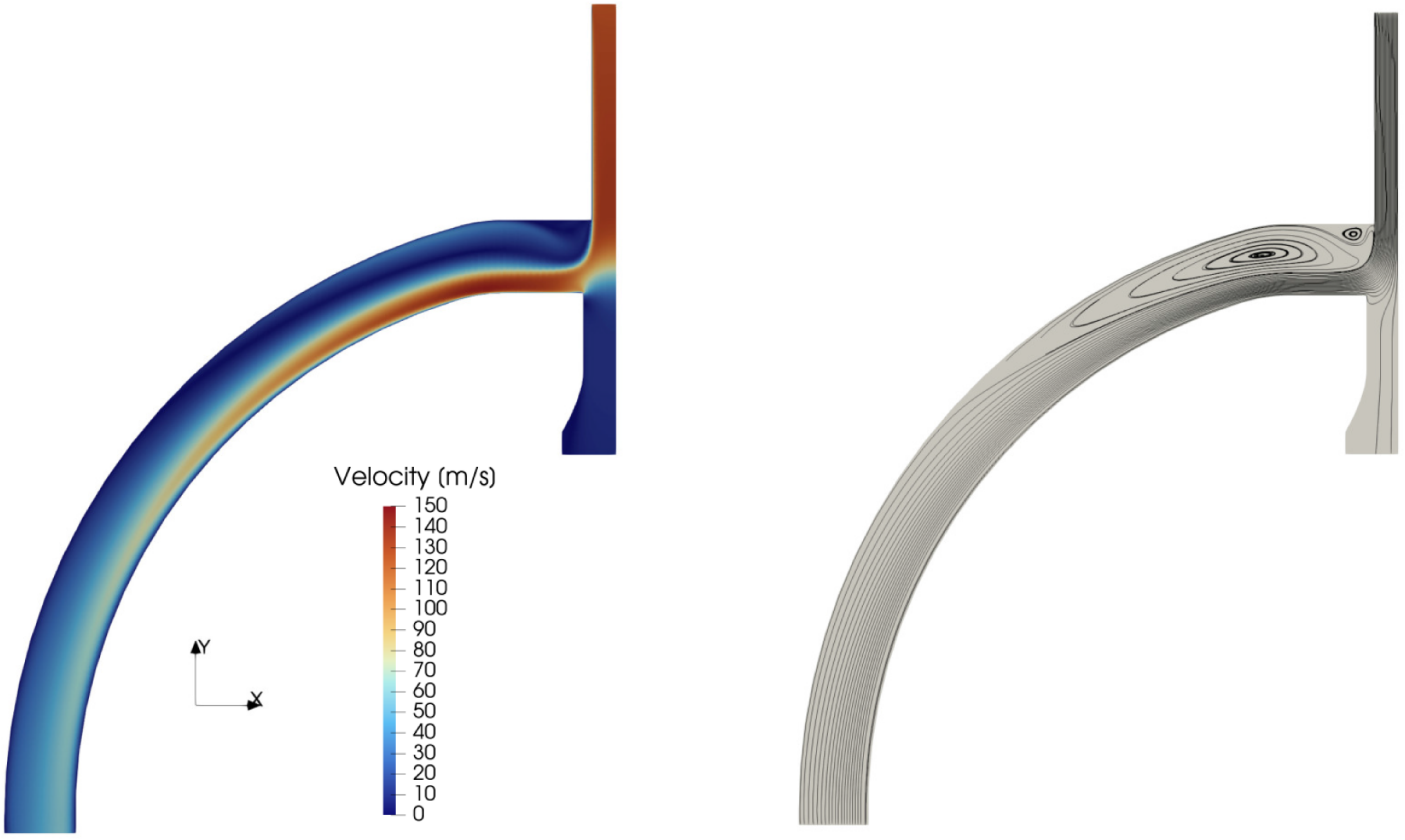
(left) Velocity field and (right) flow streamlines of the 1 μm cutoff diameter junction.

This junction also generates eddies near the inner corner of the major flow channel; a large, flattened eddy rotating clockwise and a much smaller one rotating counterclockwise.

#### Lagrangian Particles

The transient particle distributions illustrate the progressive establishment of the separation process within the virtual impactor. At early times (Figure 8a–b), particles are confined to the inlet channel and remain relatively uniformly distributed, except for a bottleneck (1 mm long) at the entrance of the acceleration nozzle that reduces its effective width by about 15% and initiates a first size-dependent separation. This bottleneck results from the observation that large particles near the wall of the acceleration nozzle tend to lose velocity and become slightly deviated upon reaching the junction, causing them to be entrained into the major flow. As a result, the major flow would experience contamination by large particles. As a remedy, the bottleneck forces large particles to detach from the wall, as their motion becomes dominated by inertia rather than by the flow streamlines. Figure 8b shows that downstream of the bottleneck, only small particles (blue) remain in close proximity to the wall, while larger particles (red/yellow) approximately maintain the distance imposed by the bottleneck.

**Figure 8:**
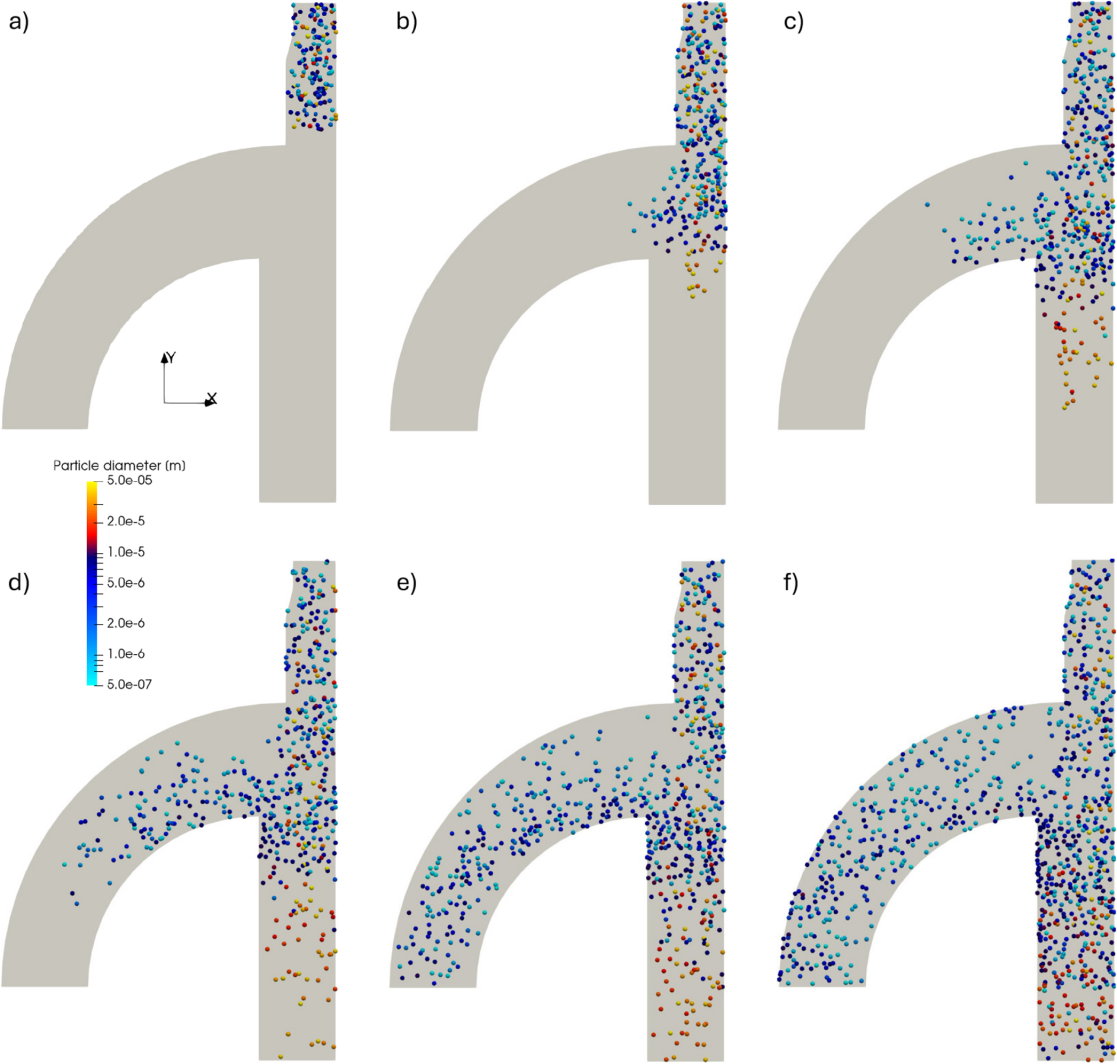
Visualization of the transient progression of the Lagrangian phase through the 10 μm junction (a–f). While particles are represented as spheres of identical size (not to scale), a color gradient indicates their diameter: blue for small particles and red/yellow for large particles. A 1 mm bottleneck at the nozzle entrance reduces the effective width by approximately 15%. (a) Particles entering the inlet channel. (b) Size-dependent separation begins at the junction: larger particles (red/yellow) continue downward due to inertia while smaller particles (blue) follow the curved streamlines toward the major flow outlet. (c) Flow topology dominates: particles are increasingly diverted into the curved branch and minor flow, with dispersion increasing near the junction. (d) The recirculation eddy at the inner corner begins to influence particle trajectories, promoting lateral spreading. (e) Clear size-dependent segregation emerges toward the major flow (left) and minor flow (downward) outlets. (f) Quasi-steady classification regime with imperfect separation; some small particles remain in the minor flow.

As particles begin to approach the junction (Figure 8b–c), a clear deviation from streamline-following behavior is seen: larger particles (red/yellow) retain higher momentum and tend to continue along the main downward direction, while smaller particles (blue) respond more strongly to local flow structures, as expected.

In the stages shown in Figure 8c–d, the influence of the flow topology becomes dominant. A significant fraction of particles is diverted into the curved branch toward the major flow outlet (left), while others enter the minor flow (downward outlet). The particle field becomes increasingly dispersed, particularly in the curved section, reflecting the combined effects of curvature-induced asymmetry and the recirculation zone previously identified in the carrier phase. This recirculation region promotes lateral spreading and a nonuniform particle distribution near the junction.

At later times (Figure 8d–f), the system approaches a quasi-steady classification regime. A clear size-dependent segregation is observed. Larger particles preferentially remain in the high-momentum core and are transported toward the minor flow outlet, while smaller particles are more readily entrained into the curved path leading to the major flow outlet. However, the separation remains imperfect. A noticeable fraction of small particles is still present in the minor flow, and some intermediate-to-large particles are dispersed into the major flow. This behavior is consistent with the presence of the recirculation zone and a nonideal dividing streamline, which introduce particle trapping, re-entrainment, and trajectory dispersion.

Figure 9 illustrates the particle classification performance of this junction. The current design has a cutoff diameter of 9.0 μm, slightly below the initial target of 10 μm. However, it was decided to stop at this stage of optimization before introducing additional complexity into the numerical model and conducting a detailed experimental validation of the results, and because this cutoff diameter is meeting the current performance requirements. The optimal cutoff diameter will be studied in later development stages, when the wall losses and the other current limits will be explored.

**Figure 9:**
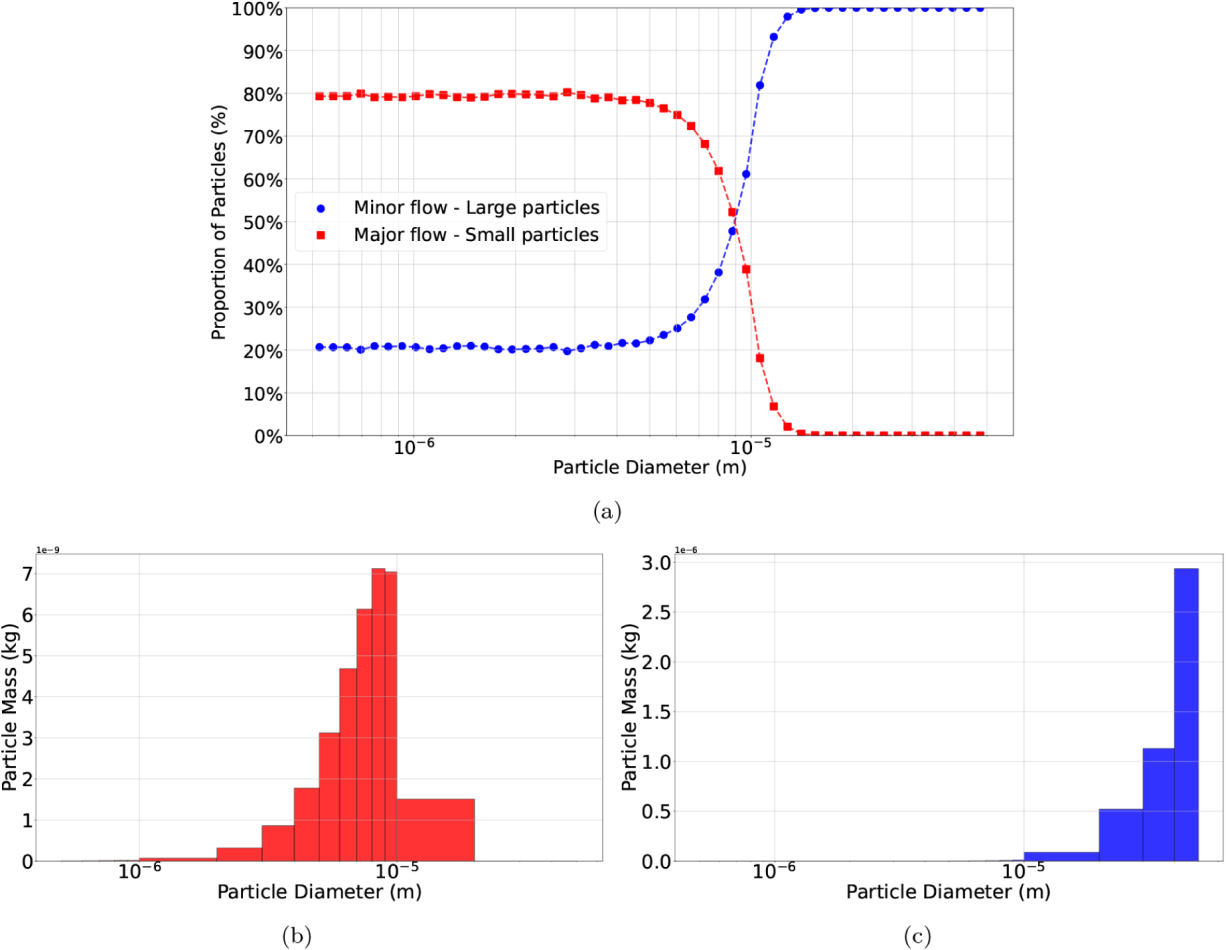
(a) Collection curves of the minor and major flows of the 10 μm junction from the numerical simulation, showing a cutoff diameter *d*_50_ = 9.0 μm and sharpness $\gamma =1.7(\sigma_{g}^{*}=1.23)$ . Small-particle contamination in the minor flow is approximately 20%. (b) Mass-based size distribution of the particles collected by the major flow and (c) the minor flow.

The performance of a virtual impactor can be evaluated by estimating the unavoidable contamination of the minor flow by small particles, the sharpness of the separation curve, and the wall losses. In the present study, the latter parameter was not evaluated, as wall interactions were modeled assuming full energy-recovery rebound and would require more advanced model benchmarking. Quantitative evaluation of wall losses through experimental measurements is planned as part of future work and will be used to implement a wall interaction numerical model.

Regarding particle contamination, Figure 9 suggests that the small-particle contamination is about 20% for this junction, and no large particles are observed beyond 20 μm in diameter. Most virtual impactors reported in the literature have small-particle contamination close to 10% (comparable to the minor-to-total flow rate ratio).^12,25^ This suggests that a limiting performance feature of the present virtual impactor has been identified and should be a key consideration for further improvements.

The origin of this reduced performance in terms of small-particle contamination is related to the intrinsic design of the μSPLIT. Small-particle contamination is inevitable in a virtual impactor due to the minor flow and primarily originates from particles located near the central streamlines directed toward the minor outlet. Traditional virtual impactors are typically cylindrical, which minimizes the extent of these central streamlines, whereas in the present flat or pseudotwo-dimensional design, central streamlines exist across the entire thickness of the junction. This suggests a direct path for improvement by reducing the thickness-to-width ratio of the junction.

However, the intended application of the μSPLIT is the assessment of radioactive airborne particles. In practice, the chemical composition of aerosol particles often varies with size due to different formation mechanisms. Nevertheless, if particles of different sizes originate from the same source and thus share the same chemical composition, their radioactivity can be assumed proportional to their mass. Consequently, the performance of the current virtual impactor should be evaluated in terms of mass rather than particle number. Figure 9b–c shows the mass distribution in each flow and demonstrates that no large-particle contamination is present in the major flow beyond 20 μm in diameter. More importantly, the mass of small particles collected in the minor flow is negligible and is therefore unlikely to significantly affect the radioactivity measurement.

Finally, the sharpness can be evaluated following several methods. Some studies evaluate the sharpness as follows^26^

15 $$\gamma =\frac{d_{p75}}{d_{p25}}$$

where *d*_*p*75_ and *d*_*p*25_ are particle diameters at 75% and 25% efficiency.

For the 10 μm junction, γ = 1.7. As a reference, some values have been extracted from research papers describing virtual impactor designs or optimizations. Numerical simulation results from previous studies suggest γ = 2.6,^15^ γ = 1.7 and γ = 1.2.^18^ Experimental results from previous studies suggest γ = 1.5 and γ = 1.3,^25^ while others calculated several values between 1.67 and 3.29.^26^ Thus, according to this metric, the current design can be considered relatively sharp, especially compared to works that develop a very similar design.^15^

Observations made at the 1 μm junction are very similar to those described previously (Figure 10). However, no bottleneck was implemented upstream of the acceleration nozzle due to the resolution limits of the 3D printer. The current width of the acceleration nozzle is 0.9 mm, meaning that an effective bottleneck would need to be approximately 0.1 mm narrower (about 50 μm on each wall) without significantly disrupting the flow.

**Figure 10:**
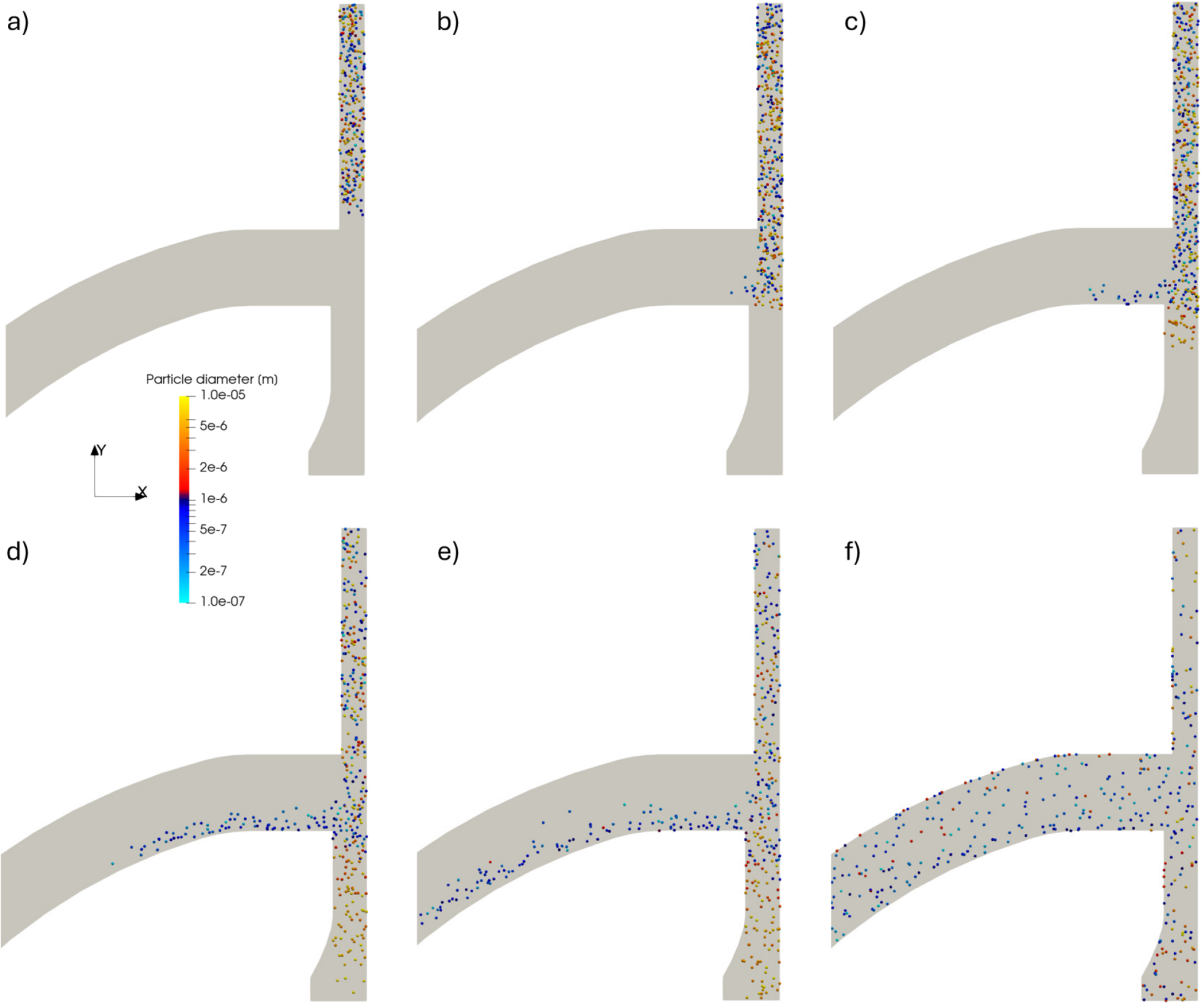
Visualization of the transient progression of the lagrangian phase through the 1 μm junction (a–f). While particles are represented as spheres of identical size (not to scale), a color gradient indicates their diameter: blue for small particles and red/yellow for large particles. No bottleneck was implemented due to printer resolution limits; the acceleration nozzle width is 0.9 mm and the flow reaches approximately 150 m/s (Ma ≈ 0.5). The geometry has been truncated for visualization purposes. (a) Particles entering the inlet channel. (b) Particles approaching the junction; larger particles (red/yellow) begin to deviate from the streamlines. (c) Size-dependent separation at the junction as particles enter the curved and minor flow channels. (d) Increased particle dispersion in the curved channel; particles follow the streamlines without significant eddy influence yet. (e) Recirculation eddies begin to capture particles. (f) Quasi-steady regime with residual particle mixing.

However, the theoretical pixel resolution of the 3D printer’s mask is 50 μm, and specifying features at this limit requires extensive prototyping, reproducibility testing, and validation. As a result, larger particles (yellow/red) can be observed near the wall of the acceleration nozzle, deviating slightly toward the left due to the curved streamlines, and impacting the bottom wall of the major flow channel near the junction between the major and minor flows, before continuing their trajectory toward the major flow outlet (Figure 10).

The cutoff diameter of the second junction was calculated to be 1.3 μm (Figure 11). This stage produces a lower contamination of small particles in the minor flow, about 13%, which is close to the target value of 10%. The origin of this discrepancy between the two junctions remains unclear. The second junction operates at much higher velocities and under compressible flow conditions, which may influence the level of contamination. In particular, it has been observed that the turbulent kinematic viscosity is significantly higher in the splitting region of the 10 μm junction compared to the 1 μm junction (Supporting Information S3). Turbulent kinematic viscosity is a good indicator of local turbulent activity, which may affect the extent of contamination and could be related to the relatively wider geometry of the 10 μm junction. It should also be noted that while the effect of Brownian diffusion on the trajectory of particles near the 1 μm cutoff is estimated to be small, it may nonetheless contribute to increased contamination of the minor flow by sub-100 nm particles.

**Figure 11:**
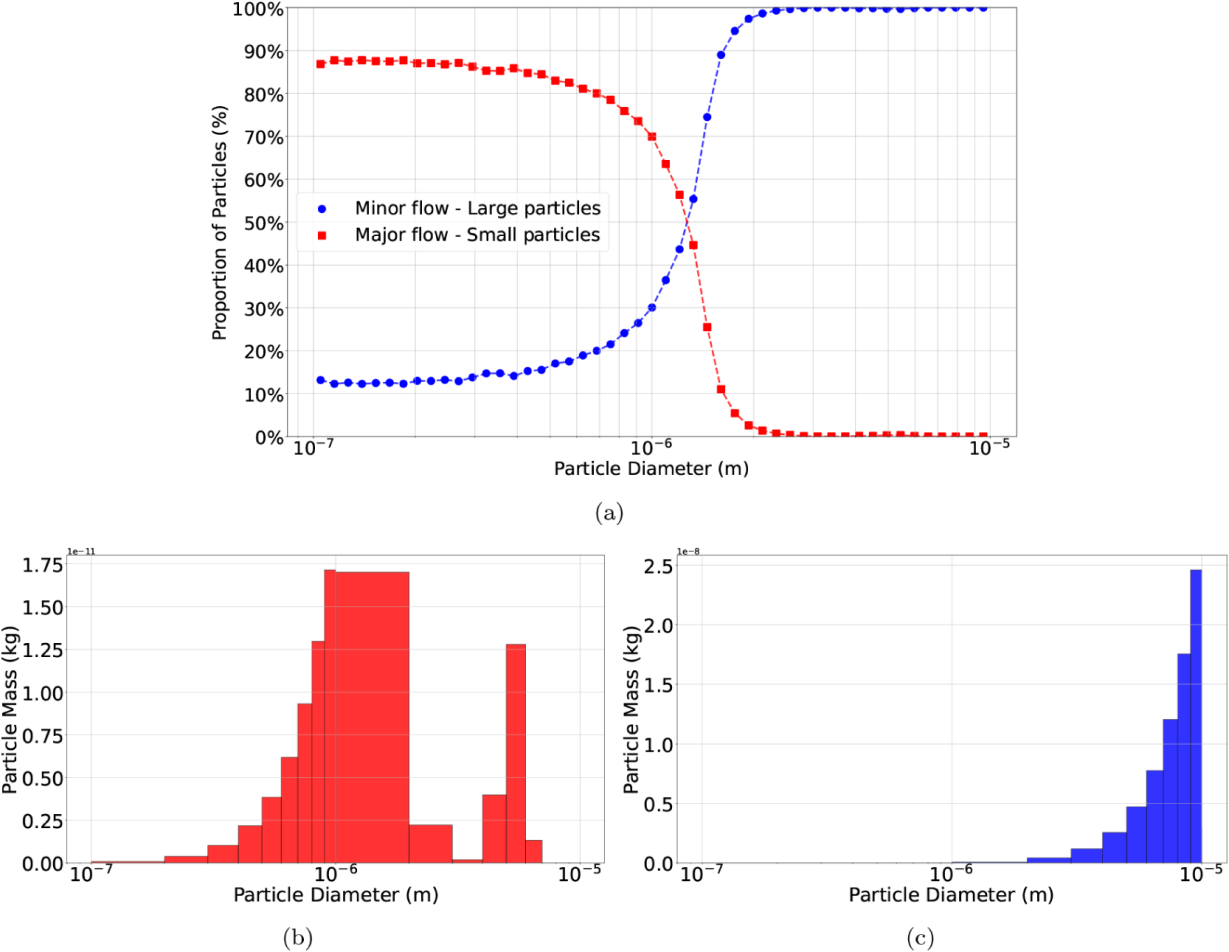
(a) Collection curves of the minor and major flows of the 1 μm junction from the numerical simulation, showing a cutoff diameter *d*_50_ = 1.3 μm and sharpness $\gamma =1.7(\sigma_{g}^{*}=1.30)$ . Small-particle contamination in the minor flow is approximately 13%. (b) Mass-based size distribution of the particles collected by the major flow, revealing significant large-particle contamination by particles of 4–7 μm, and (c) the minor flow.

The sharpness of the 1 μm junction is γ = 1.7, similar to that of the previous stage. However, a closer examination reveals that, due to the low contamination at smaller diameters, the separation curve of the second junction appears significantly less sharp on the left side of the cutoff diameter.

The efficiency curve of inertial impactors can be approximated by a log-normal distribution of the particle diameter.^27^ Consequently, the geometric standard deviation can be used as an additional metric to quantify the sharpness.

16 $$\sigma_{g}=\sqrt{\frac{d_{84}}{d_{16}}}$$

where *d*_84_ and *d*_16_ are the particle diameters at 16% and 84% collection (chosen to correspond to one standard deviation). If σ_*g*_ = 1, the curve is a perfect step function.

However, collection curves do not reach 0% due to the small particle contamination. Consequently, to evaluate purely the curve profile, $\sigma_{g}^{*}$ is defined as

17 $$\sigma_{g}^{*}=\sqrt{\frac{d_{84}^{*}}{d_{16}^{*}}}$$

where $d_{84}^{*}$ and $d_{16}^{*}$ are the corrected particle diameters at 16% and 84% of the collection relative to the asymptote of contamination.

The current corrected standard deviation values are $\sigma_{g,10\mu m}^{*}=1.23$ and $\sigma_{g,1\mu m}^{*}=1.30$ . As a reference, the corrected standard deviation was calculated previously as $\sigma_{g,Loo\&Cork}^{*}=1.16$ .^25^ This suggests that the current numerical simulations predict a slightly lower sharpness than that of a highly optimized experimental virtual impactor, particularly for the second junction.

Figure 11b highlights a major issue with this second junction. The major flow produces significant contamination by large particles (4 to 7 μm). Although the number of such particles is low, their larger size and mass mean they can significantly affect the activity measurement.

Figure 12a shows the trajectories of particles with diameters greater than 2 μm that exit the domain through the major flow outlet. This confirms that particles that should exit through the minor flow outlet are misclassified due to their proximity to the inner wall of the acceleration nozzle. In fact, these particles come into contact with the wall and lose part of their velocity in the process. Upon reaching the separation junction, their reduced momentum makes their trajectories more sensitive to the curved streamlines, leading them to impact the bottom wall of the major flow channel.

**Figure 12:**
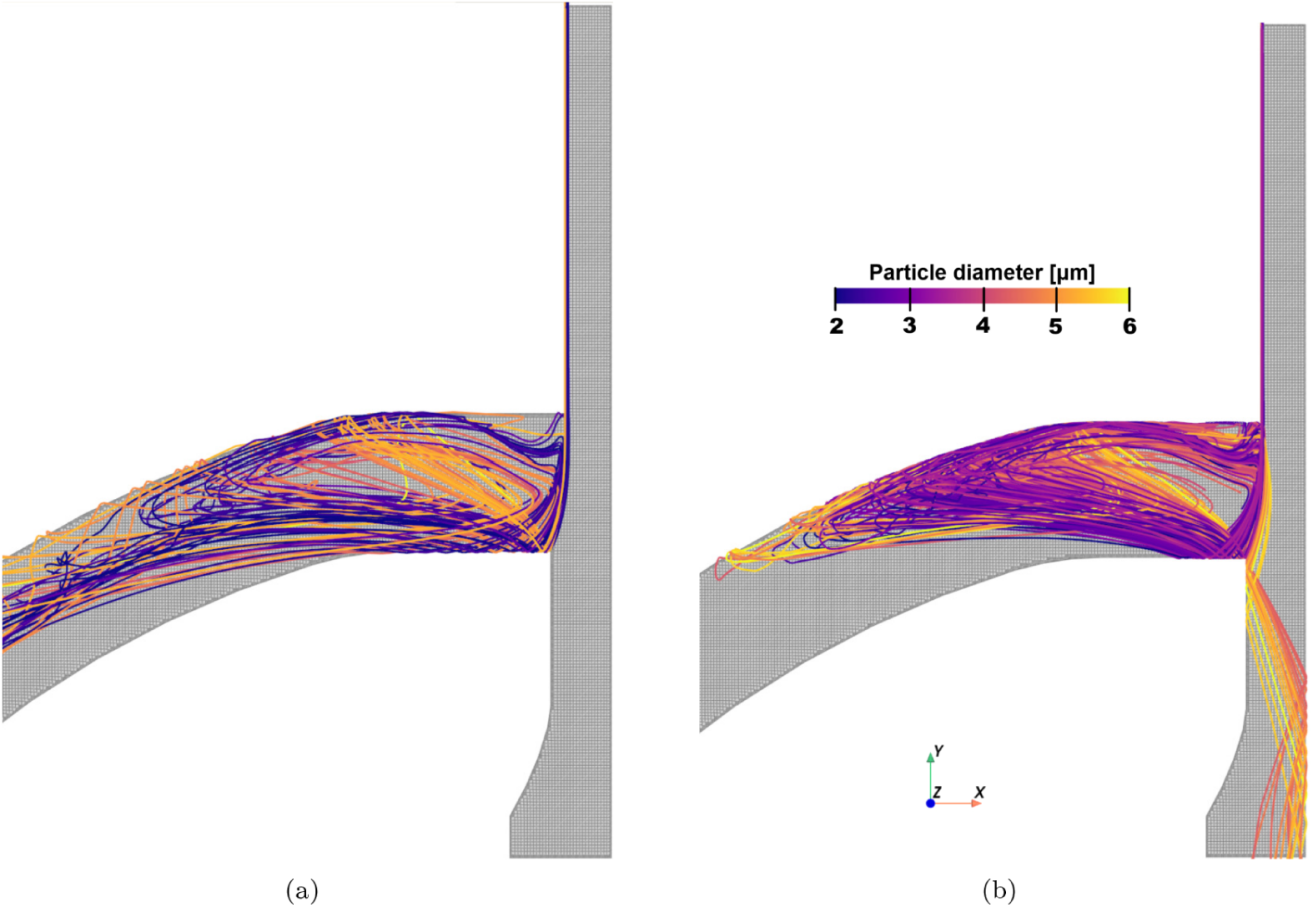
(a) Trajectories of particles larger than 2 μm that are misclassified into the major flow outlet, showing wall-contact-induced momentum loss as the primary cause. (b) Trajectories of particles larger than 2 μm that temporarily enter the major flow channel: particles of 4–7 μm are partially reinjected into the minor flow via the recirculation eddy, while particles of 2–4 μm remain trapped in a persistent cycle without successful reinjection.

In contrast, Figure 12b shows the trajectories of particles with diameters greater than 2 μm that temporarily enter the major flow channel but do not exit through the major flow outlet. A closer inspection indicates that particles larger than 4 or 5 μm can interact with the recirculation eddy, and some are reinjected into the junction and eventually exit through the minor flow outlet, as expected. However, particles in the 2 to 4 μm range appear to be trapped in a persistent cycle induced by the eddy, without successful reinjection into the minor flow. These particles are eventually removed numerically by the solver, which explains their lower yield in Figure 11b. In practice, such particles would likely contribute to wall losses.

Overall, these particles share a common feature: contact with, or close proximity to, the inner wall of the acceleration nozzle. The limitations imposed by the current printing resolution should therefore be thoroughly investigated through prototyping to mitigate this behavior and eliminate large-particle contamination, as achieved for the first junction. A parametric study based on systematic STL geometry variations, including nozzle width, wall taper angle, and junction curvature is planned to further investigate which design modifications most effectively suppress large-particle contamination in the second stage.

An additional strategy is proposed for future improvements to address large particle contamination and, potentially, small particle contamination. Early developments in experimental precision virtual impactors demonstrated that a layer of clean air could be injected into the acceleration nozzle near the walls, effectively isolating the particle-laden flow from the walls by means of a clean air sheath.^17^ Under such conditions, wall effects would be rendered negligible, thereby preventing significant velocity losses in large particles that would otherwise lead to their misclassification. This modification would necessitate an additional clean air inlet and further flow path engineering.

### Prototype Printing

#### Prototype Description

The model was printed vertically, with a minimal amount of temporary support to reduce surface alteration and limit manual postprocessing. The prototypes were printed using the smallest available layer thickness (0.025 mm). The printer can accommodate 15 μSPLIT at a time, and the total printing duration is about 20 h. Additional tests will be conducted to assess whether a coarser layer resolution impacts prototype quality, as it could reduce the printing time by a factor of 10.

The inlet and outlets of the μSPLIT can be threaded to accept M12 (ISO) gas connectors or printed with inner diameters of 6 and 8 mm to allow insertion and bonding of flexible tubing. As shown in Figure 5a, the top plane includes three openings to allow insertion and removal of filtration membranes, as well as direct measurement of α-emitters. After printing and filter insertion, the μSPLIT must be sealed to ensure airtightness. This is achieved by placing a thin polyester film (Mylar, 4 μm thick) over the three filter openings and bonding it with a two-component epoxy adhesive. The Mylar film was selected for its good transmission of α-particles and its mechanical strength (Figure 13).

**Figure 13:**
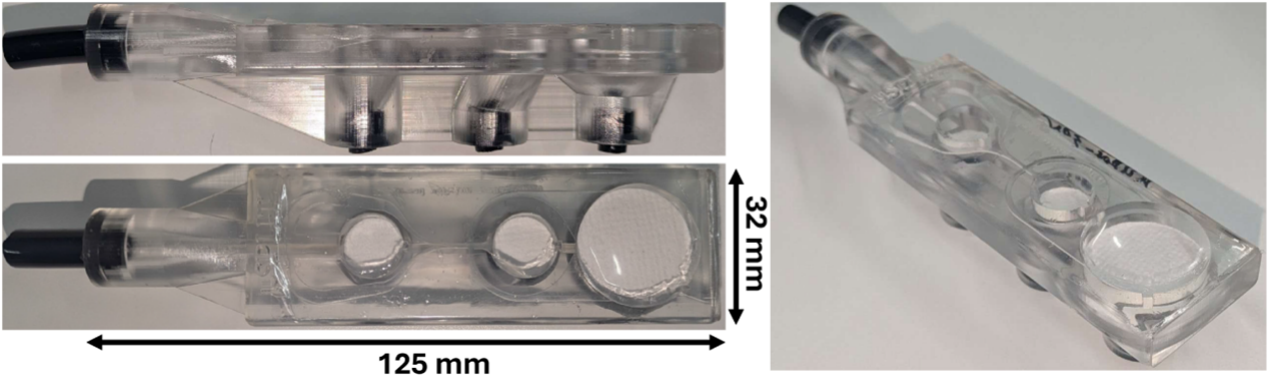
Photos of the μSPLIT printed with the clear resin. This version has flexible pipes inserted and glued in the inlet and outlets, filters installed and the Mylar film stuck. Residual epoxy glue and reflections can attest to the Mylar film presence.

#### Acceleration Nozzle Measurement

Due to the fabrication process, 3D-printed prototypes tend to shrink during curing. To quantify this effect, the internal flow path was measured using a digital microscope (Keyence VHX-X1). Measurements were performed on three different prototypes printed over several months to assess the reproducibility of the printing process. The prototypes were sectioned using a bandsaw.

Table 1 presents measurements of the height and width of the acceleration nozzles for three μSPLIT devices from different batches produced over several months. A consistent contraction relative to the original specifications is observed, likely due to curing effects. Prototype no 3 exhibits the largest shrinkage, with three out of four measured dimensions exceeding a 4% deviation and reaching up to 6.8% contraction. Such deviations from the design specifications may lead to significant changes in the cutoff diameter. The current objective is to remain within a ±2% deviation, although a more detailed analysis is required to define acceptable tolerances more precisely.

**Table 1:** Height and Width of the Acceleration Nozzle Channels of the Two Junctions of Three μSPLITs Measured Using a Digital Microscope

	10 μm junction		1 μm junction	
Prototype	Height [μm]	Width [μm]	Height [μm]	Width [μm]
Specifications	3500	3500	1000	900
no 1	3458 (−1.2%)	3497 (−0.1%)	981 (−1.9%)	879 (−2.4%)
no 2	3501 (∼ 0%)	3481 (−0.5%)	984 (−1.6%)	861 (−4.5%)
no 3	3341 (−4.8%)	3474 (−0.7%)	960 (−4.2%)	843 (−6.8%)

Following this assessment, several actions have been defined to limit deviations from the specifications: (i) regarding the design, the prototypes will be printed at 102% of their nominal dimensions to compensate for shrinkage; (ii) their position on the printing bed will be kept identical from one print to another to ensure reproducibility; and (iii) after complete curing, a nondestructive quality check will be performed by measuring the width of selected channels (e.g., acceleration nozzles) using a digital microscope, taking advantage of the transparent resin.

Microscope images shown in Figure 14 indicate that the walls are slightly curved and that the corners are significantly rounded. This is attributed to the surface tension of residual liquid resin accumulating in the corners. The current cleaning process relies on an agitated isopropanol bath, but it could be improved by actively flushing isopropanol through the channels of the μSPLIT using an electric pump to enhance the removal of residual resin prior to final curing. However, most sharp edges will inevitably be rounded to a certain extent.

**Figure 14:**
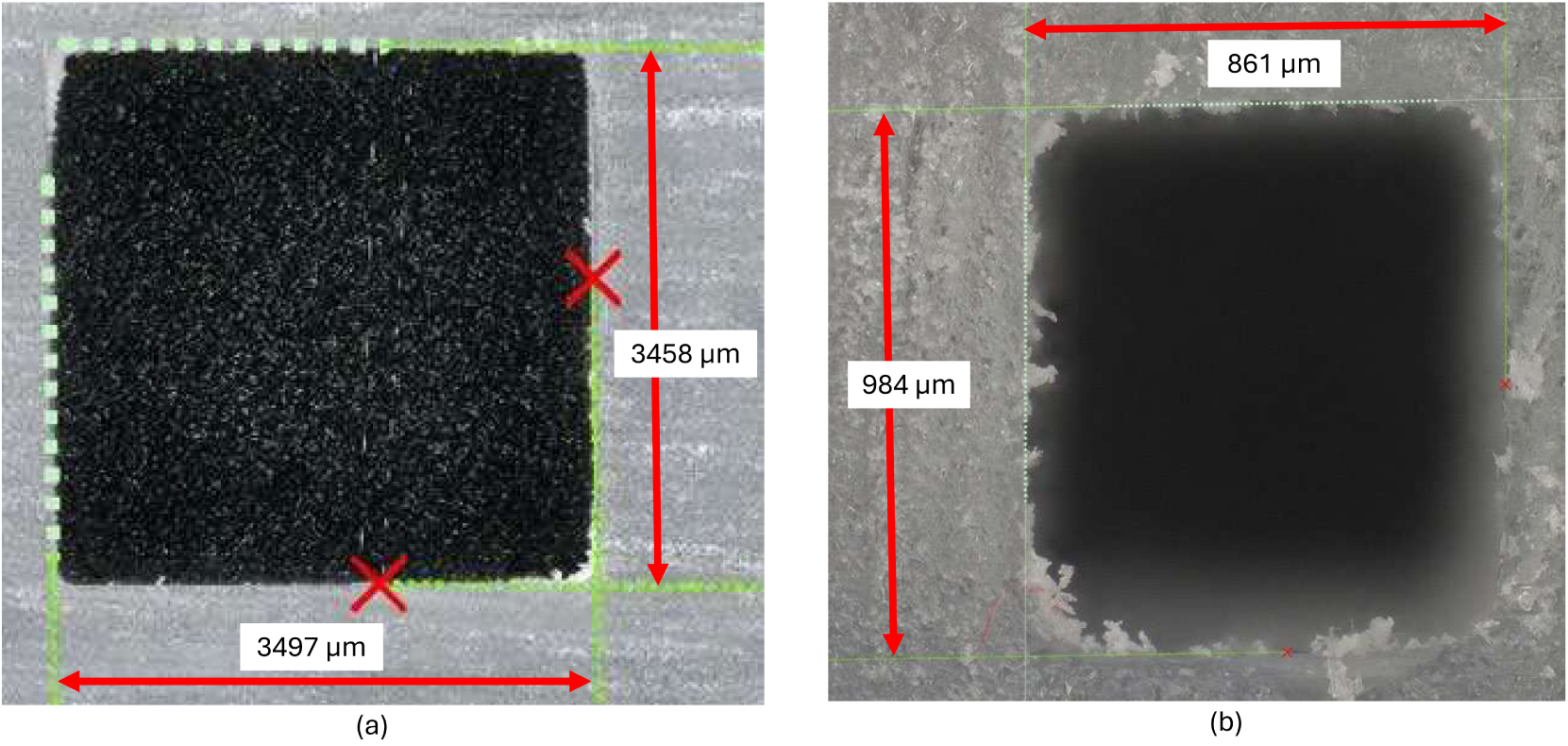
Digital microscope images of slices of μSPLITs, perpendicular to the flow channels. (a) is an image of the first junction’s acceleration nozzle and (b) is the acceleration nozzle of the second junction.

## Experimental Results

### Incense Smoke Collection

This experiment was conducted as a qualitative functional check of the prototype’s ability to handle and classify submicron aerosols, rather than as a quantitative validation of the cutoff diameters. Figure 15a illustrates the size distribution of incense smoke particles accumulated prior to injection through the μSPLIT. These particles are predominantly submicrometric in aerodynamic diameter, with the highest mass fraction in the 100 nm to 1 μm range (peak in the 317 to 594 nm bin). This is consistent with previous measurements of mass-based aerodynamic size distributions.^28–30^

**Figure 15:**
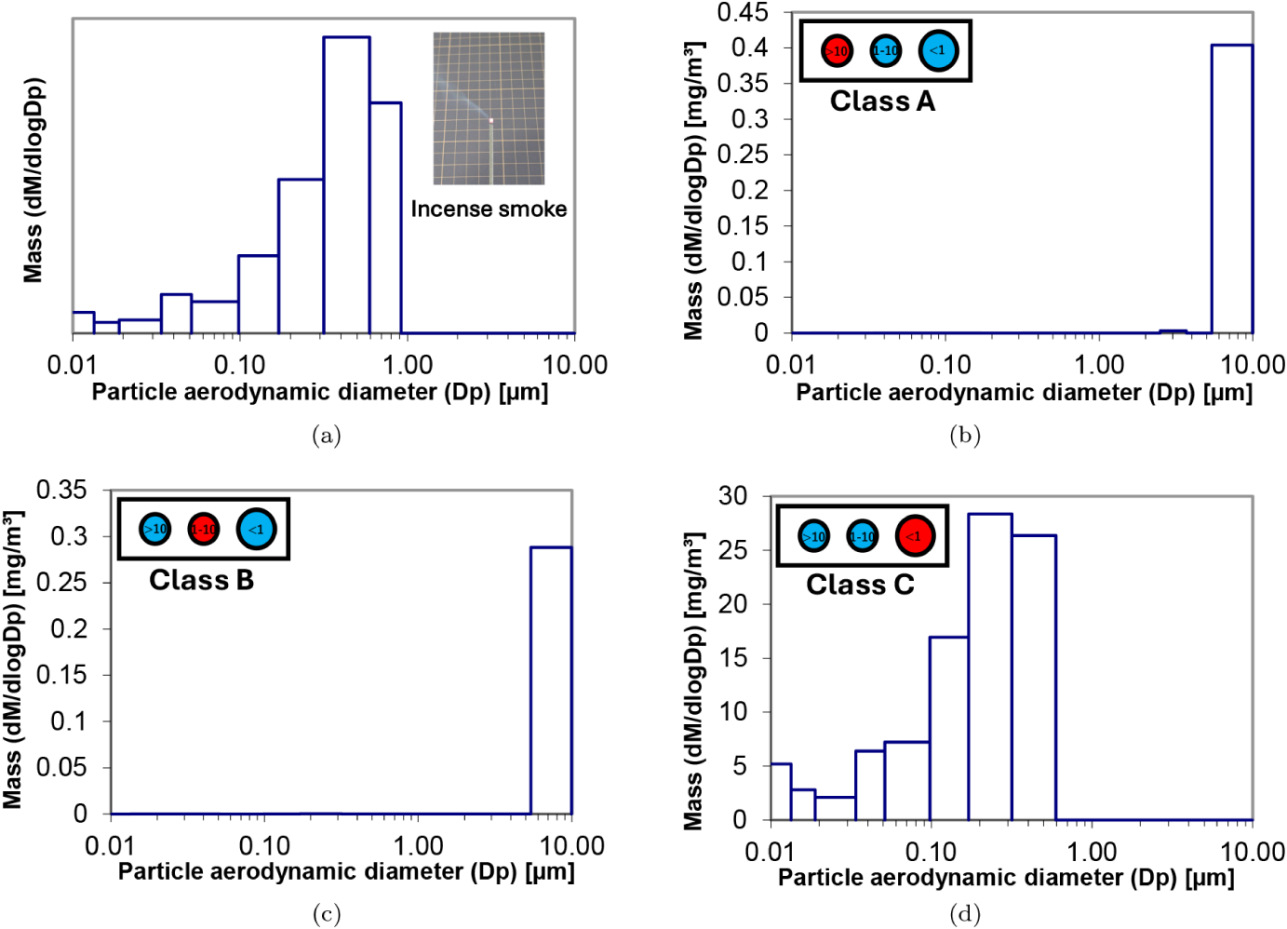
(a) Mass-based particle aerodynamic diameter distribution of accumulated incense smoke. (b–d) Resulting mass-based particle aerodynamic diameter distribution of the incense smoke after going through the μSPLIT and being collected at the class A, class B, and class C outlets, respectively.

The mass size distributions obtained after sampling and classification by the μSPLIT are shown in Figure 15b–d. The collected mass for class A and class B particles is approximately 2 orders of magnitude lower than that for class C particles and can be considered negligible. In particular, the only visible contribution corresponds to the largest size bin, which is attributed to background noise.

The size distribution of the collected class C particles is broadly similar to that of the injected incense smoke. A slight discrepancy is observed: class C particles show negligible mass in the 594 to 916 nm bin and comparable mass in the 170 to 317 nm and 317 to 594 nm ranges, whereas the second-highest bin in the initial incense distribution is the 594 to 916 nm range, and the 317 to 594 nm bin is approximately twice as high as the 170 to 317 nm bin. However, since the collected mass of class B particles is negligible, this difference is likely due to wall deposition near the junction. This interpretation is consistent with the numerical simulations, which indicate that recirculation zones can promote the trapping of particles with diameters close to the cutoff and lead to their deposition on the walls. In addition, a black deposit was observed on the walls near the second junction after the experiment, which may result from this mechanism, although its particle size distribution could not be determined.

### ^222^Rn-NaCl Particles Collection

Prior to conducting the experiments, the mass-based size distribution of the dried and solidified NaCl particles was measured and is presented in Figure 16a. The particle size distribution is well described by a unimodal log-normal function, characterized by a geometric mean diameter of 1.70 μm and a geometric standard deviation of 1.76. In other words, particles are expected to predominantly fall into class B and accumulate on the second filter.

**Figure 16:**
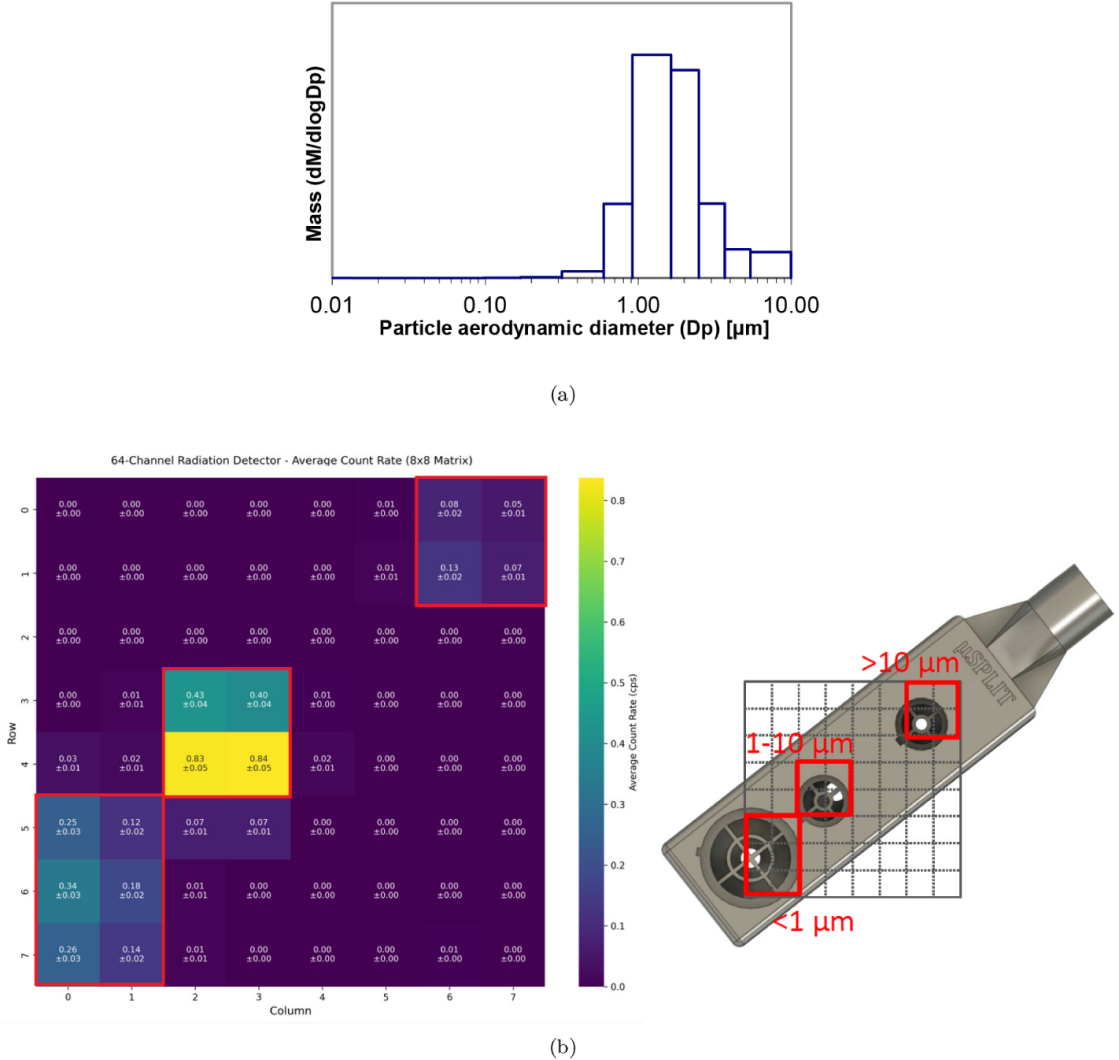
(a) Mass-based Rn-progeny-bearing NaCl particle aerodynamic diameter distribution. (b) Heatmap of the count rate per second and per pixel of the α-particle detector averaged over 300 s and placed in contact with the upper face of the μSPLIT after the experiment (±1 σ error). The position of the detector relative to the μSPLIT is also shown.

The α-particle detector consists of an 8 × 8 pixel array (with a pitch of ∼6.4 mm) and was positioned diagonally so that the three filters could at least partially irradiate the detection area. The average count rate per second (cps) per pixel is shown in Figure 16b over a 300 s measurement (average value), along with the position of the detector relative to the μSPLIT. The count rate for the four pixels exposed to the class A filter is low, ranging from 0.05 to 0.13 cps; for the four pixels exposed to the class B filter, it is the highest, ranging from 0.40 to 0.84 cps; and for the six pixels exposed to the class C filter, it is intermediate, ranging from 0.12 to 0.34 cps. It should be noted that pixels with the lowest count rates may only be partially exposed, meaning that the highest values are likely more representative.

These results support the expected classification performance. Although the current size spectrometer does not allow reliable measurement of particles larger than 10 μm, it confirms that the generated α-emitting aerosol particles are predominantly collected on the class B filter. This is particularly notable given that the mass flow rate through the class C filter is eight times higher than that through the class B filter, while the filter area is only 3.7 times larger.

At present, a fully quantitative comparison remains challenging for the following considerations. If radioactivity were simply proportional to particle mass, the α count rate on the class B filter (1–10 μm) would be expected to substantially exceed that on the class C filter (<1 μm), given that the mass-based size distribution of the carrier aerosol (GMD_*m*_ = 1.70 μm, GSD = 1.76) peaks within the class B range. However, the measured α count rate on the class C filter (0.12–0.34 cps) is comparable to that on class B (0.40–0.84 cps), indicating a higher specific activity (counts per unit mass) in the fine fraction.

This observation can be explained by the mechanism of ^222^Rn progeny attachment to carrier aerosol surfaces. Radon progeny (^218^Po, etc.) are initially formed as molecular clusters of approximately 1 nm and subsequently attach to existing aerosol particles by Brownian diffusion. This attachment process is governed by the available surface area of the carrier particles rather than by their mass.^31^ The surface-area-based geometric mean diameter, GMD_*s*_, can be derived from the mass-based values using the Hatch–Choate relationship:

18 $${\mathrm{GMD}}_{s}={\mathrm{GMD}}_{m}\times \exp\left(-2\ln^{2}\mathrm{GSD}\right)$$

Substituting the measured values (GMD_*m*_ = 1.70 μm, GSD = 1.76) yields GMD_*s*_ ≈ 0.90 μm, which falls within the class C range (<1 μm). This implies that the surface area available for radon progeny attachment peaks in the fine particle fraction, consistent with the higher specific activity observed on the class C filter.

Furthermore, the large-particle contamination identified in the second-stage CFD results (Figure 11b) may contribute additional activity to the class C stage, as high-activity intermediate-size particles are diverted into the fine-particle collection path. Consequently, a fully quantitative comparison would require (i) direct measurement of the surface-area distribution of the carrier aerosol, (ii) correction for the misclassification effect, and (iii) verification of particle uniformity across the filter surfaces, all of which are reserved for future investigation. It should be noted that the present experiment using externally attached radon progeny represents a surface-area-governed activity distribution, which differs from the case of intrinsically radioactive particles expected in actual field conditions (e.g., fuel debris or uranium-containing aerosols), where radioactivity is more directly proportional to particle mass. The μSPLIT is intended for both scenarios, and the relative contribution of each mechanism should be assessed in the context of the specific aerosol source.

## Conclusions

This study presents the early development of a two-stage, 3D-printed virtual impactor, the μSPLIT, for the size classification and direct analysis of radioactive aerosols in challenging environments such as the Fukushima Daiichi decommissioning site. Numerical simulations using a 2D RANS approach with Lagrangian particle tracking predicted cutoff diameters of 9.0 and 1.3 μm for the first and second stages, respectively, with sharpness ( $\sigma_{g}^{*}=1.23$ and 1.30) comparable to previously reported virtual impactors. The simulations also identified design-specific limitations: small-particle contamination (20%) in the minor flow attributable to the pseudo-2D geometry, and, more notably, large-particle contamination in the major flow of the second stage caused by particle-wall interactions near the acceleration nozzle.

Prototype fabrication by stereolithography demonstrated the feasibility of a lightweight, low-cost, and potentially disposable device. Dimensional measurements of three prototypes from separate batches revealed consistent shrinkage (up to 6.8%) and corner rounding in the internal channels, underscoring the need for fabrication compensation (e.g., 102% scaling) and quality control. Experimental tests with incense smoke provided a qualitative functional check of the complete aerosol handling pathway, while tests with ^222^Rn-progeny-bearing NaCl particles (geometric mean 1.70 μm) produced the highest α activity on the class B filter, consistent with the expected classification.

Future work will focus on quantitative validation of classification efficiency using monodisperse standard particles, extension of CFD simulations to full 3D, parametric studies of STL-based geometry variations to suppress large-particle contamination, experimental quantification of wall losses, incorporation of Brownian motion effects into the particle model, and further investigation of the relationship between aerosol particle properties and radioactivity for improved interpretation of in situ measurements.

## Supplementary Material



## Notes

Section S1: additional information on the fluid model; Section S2: injected particle size distributions; Section S3: local turbulent kinematic viscosity comparison between junctions (PDF)
