# Supplementary material for: Development of a 3D-Printed Two-Stage Virtual Impactor for Radioactive Aerosol Size Classification and Direct Analysis
Source: ACS Omega. 2026 Jul 19;11(30):45423–38. doi: 10.1021/acsomega.6c04521 (PMC13448912; doi:10.1021/acsomega.6c04521)

Supplementary information

Development of a 3D-Printed Two-Stage Virtual Impactor for Radioactive Aerosol Size Classification and Direct Analysis

Hugo Laffolley[1], Youichi Tsubota[1], Ayame Kuroe[2], Tomoaki Kato[1]*

[1]Collaborative Laboratories for Advanced Decommissioning Science, Japan Atomic Energy Agency, 2-4 Shirakata, Tokai-mura, Naka-gun, Ibaraki, 319-1195, Japan

[2]Radiation Protection Department, Nuclear Cycle Engineering Laboratories, Japan Atomic Energy Agency, 4-33 Muramatsu, Tokai-mura, Naka-gun, Ibaraki, 319-1194, Japan

*Email: tsubota.yoichi@jaea.go.jp

S.1. Additional information on the fluid model:

The working fluid was modeled as a pure gas mixture representing air, using OpenFOAM's *hePsiThermo* thermodynamic package. The fluid properties were defined as follows: molecular weight M = 28.9 g mol^{-1}, specific heat capacity at constant pressure C_p = 1005 J kg^{-1} K^{-1}, and formation enthalpy Hf = 0 J kg^{-1}. Viscosity was computed via Sutherland's law with constants A_s = 1.4792 × 10^{-6} Pa s K^(1/2) and T_s = 116 K. The ideal gas equation of state (p = ρRT) was employed, assuming constant C_p and a sensible - enthalpy formulation, which is suitable for non-reacting compressible flows.

S.2. Injected particles size distribution

Figure S1.

Size distribution of particles injected in the numerical simulation for the development of the 10 μm junction.

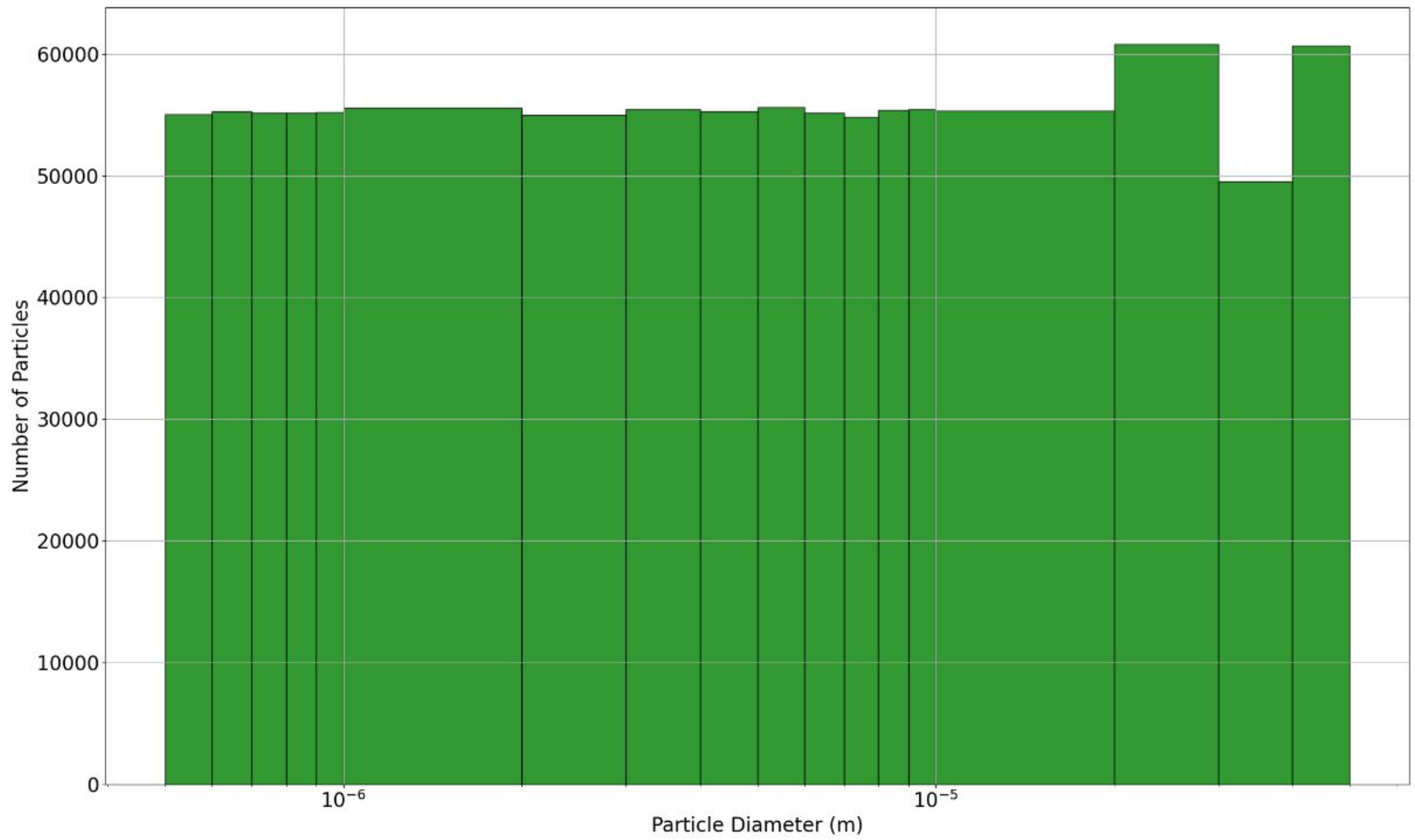

Size distribution of particles injected in the numerical simulation for the development of the 1 μm junction.

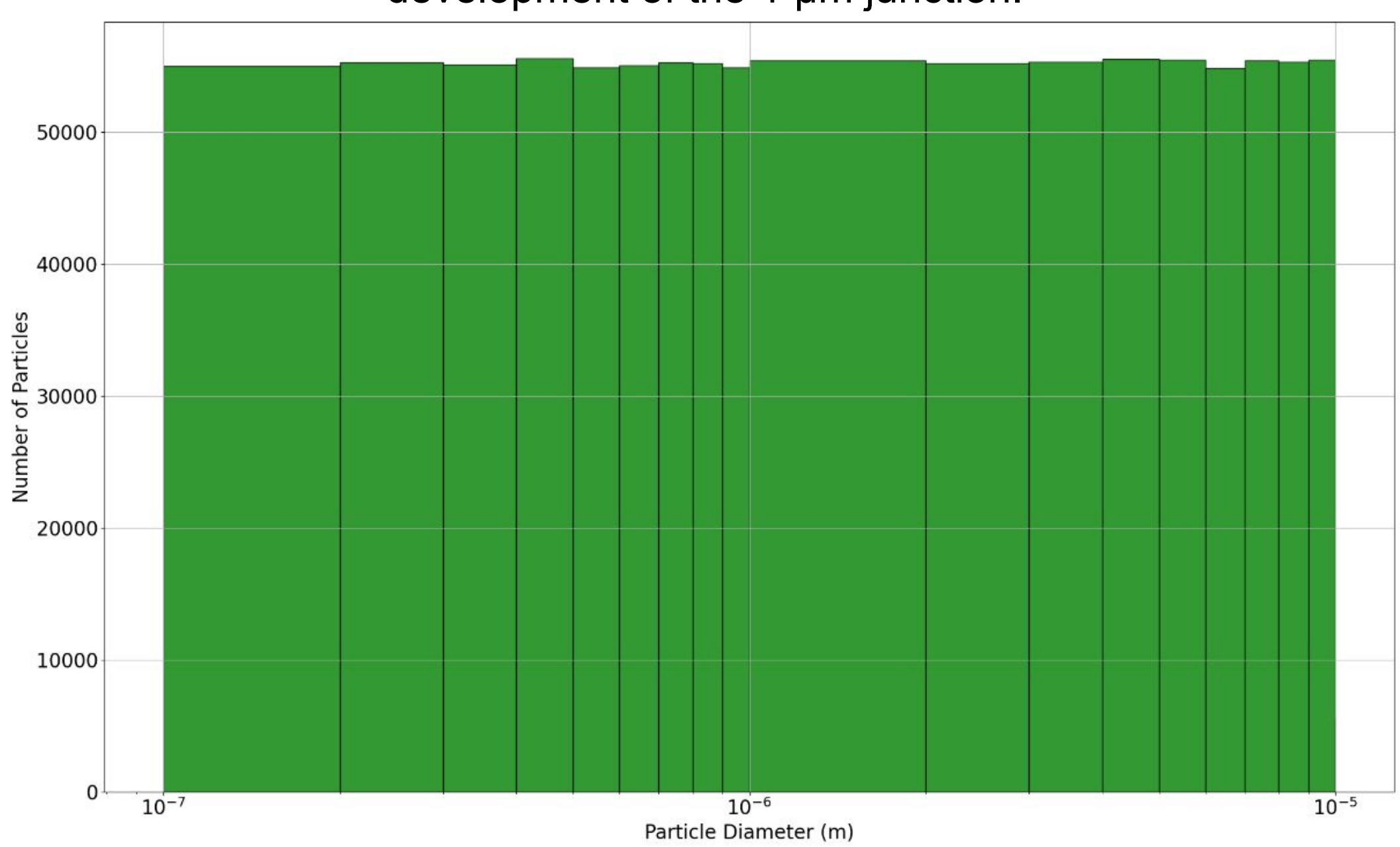

S.3. Local turbulent kinematic viscosity field showing higher values in the junction region for the first separation stage, indicating a potential contribution to the greater small particles contamination in the minor flow.

Figure S2.

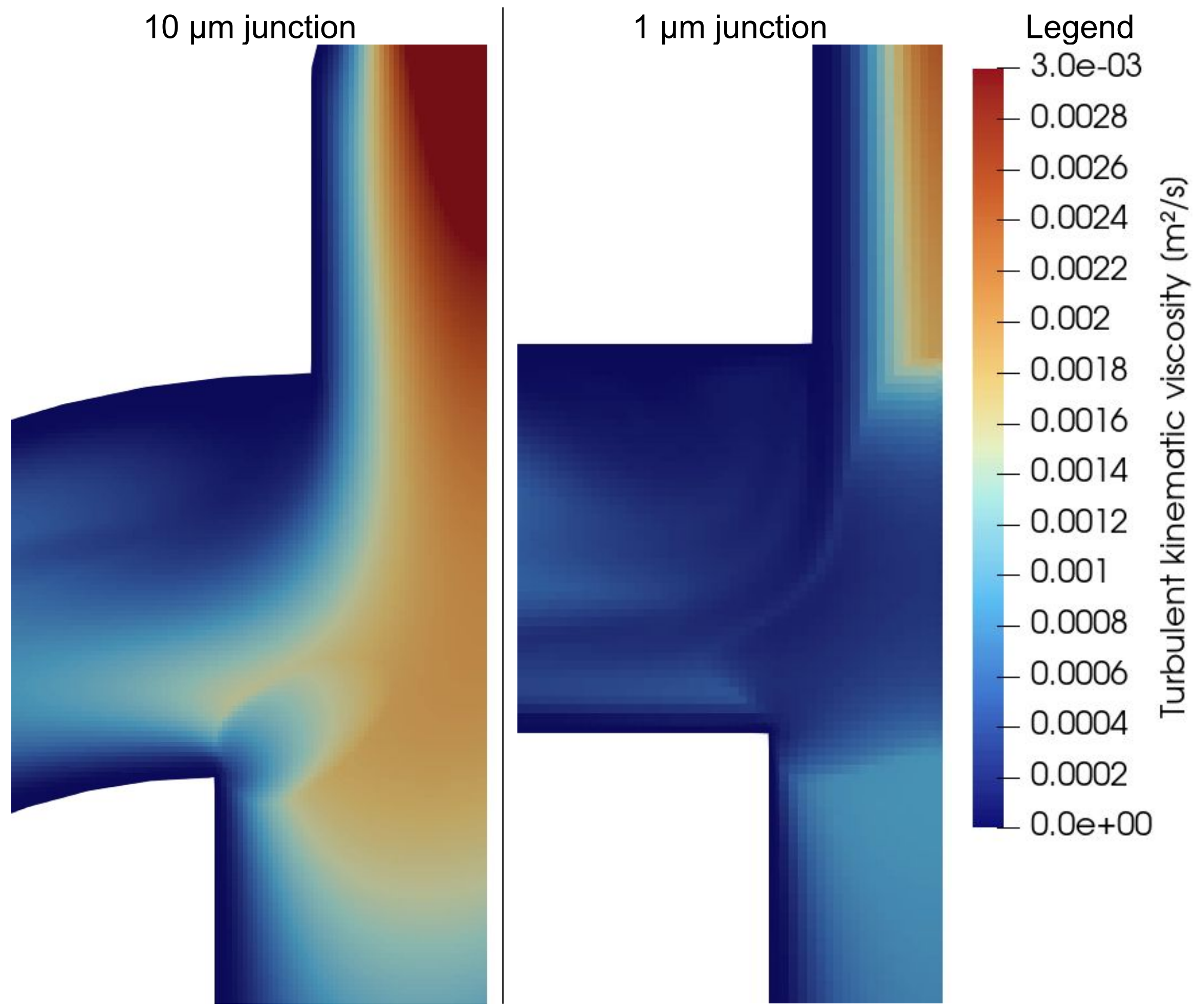

Supplement: Supplementary file 1 [file ao6c04521_si_001.pdf]
